# ConFiG: Contextual Fibre Growth to generate realistic axonal packing for diffusion MRI simulation

**DOI:** 10.1016/j.neuroimage.2020.117107

**Published:** 2020-10-15

**Authors:** Ross Callaghan, Daniel C. Alexander, Marco Palombo, Hui Zhang

**Affiliations:** Centre for Medical Image Computing and Department of Computer Science, University College London, London, UK

**Keywords:** Phantom generation, White matter, Diffusion MRI, Simulation, CAM, cell adhesion molecules, CC, corpus callosum, ConFiG, contextual fibre growth, dMRI, diffusion MRI, EM, electron microscopy, FOD, fibre orientation distribution, HCP, human connectome project, IC, internal capsule, TC, three crossing region, WM, white matter

## Abstract

This paper presents Contextual Fibre Growth (ConFiG), an approach to generate white matter numerical phantoms by mimicking natural fibre genesis. ConFiG grows fibres one-by-one, following simple rules motivated by real axonal guidance mechanisms. These simple rules enable ConFiG to generate phantoms with tuneable microstructural features by growing fibres while attempting to meet morphological targets such as user-specified density and orientation distribution. We compare ConFiG to the state-of-the-art approach based on packing fibres together by generating phantoms in a range of fibre configurations including crossing fibre bundles and orientation dispersion. Results demonstrate that ConFiG produces phantoms with up to 20% higher densities than the state-of-the-art, particularly in complex configurations with crossing fibres. We additionally show that the microstructural morphology of ConFiG phantoms is comparable to real tissue, producing diameter and orientation distributions close to electron microscopy estimates from real tissue as well as capturing complex fibre cross sections. Signals simulated from ConFiG phantoms match real diffusion MRI data well, showing that ConFiG phantoms can be used to generate realistic diffusion MRI data. This demonstrates the feasibility of ConFiG to generate realistic synthetic diffusion MRI data for developing and validating microstructure modelling approaches.

## Highlights

• We present ConFiG, a biologically motivated numerical phantom generator for white matter.
• ConFiG produces phantoms with state-of-the-art density and realistic microstructure.
• Diffusion MRI simulations in ConFiG phantoms are comparable to real dMRI signals.

## 1 Introduction

Numerical phantoms play a valuable role in the development and validation of many magnetic resonance imaging (MRI) techniques. In particular, numerical phantoms are often used when developing diffusion MRI (dMRI) microstructure imaging techniques where simulations of the dMRI signal in phantoms with known microstructural properties are used in lieu of an *in vivo* ground truth measure of microstructure (Alexander et al., 2017). While recently numerical phantoms have proven useful for validating microstructure imaging of grey matter (Palombo et al., 2020), they have more commonly been used for validating white matter (WM) microstructure, with many studies comparing parameter estimates from fitting their models to the known ground truth from the phantoms e.g. (Daducci et al., 2015; Jelescu and Budde, 2017; Li et al., 2019; Nilsson et al., 2017, 2010; Scherrer et al., 2016; Tariq et al., 2016; Xu et al., 2014; Zhang et al., 2012). Some recent works directly estimate microstructural features using fingerprinting techniques and machine learning to match simulated signals and the corresponding ground truth microstructure of the numerical phantom to the measured signal (Hill et al., 2019; Nedjati-Gilani et al., 2017; Palombo et al., 2018a; Rensonnet et al., 2018). As well as affecting the dMRI signal, microstructural features also influence other MR techniques such as susceptibility-weighted imaging (Lee et al., 2010; Li et al., 2012). For instance, Xu et al. (2018) recently used simulations to show that using realistic axonal models rather than simple circular cylinders affects the MR signal. Therefore, it is important to the MRI community to generate realistic WM numerical phantoms which accurately capture microstructural features in order to get realistic simulated signal.

Typically, however, there is a mismatch between the complexity of true brain tissue microstructure and the models used in simulation, with simulations simplifying the microstructure. On one hand, ex vivo electron microscopy (EM) studies have revealed the high complexity of real axonal morphology (Abdollahzadeh et al., 2019; Lee et al., 2019; Salo et al., 2018). Reconstructions of axons from these studies show that real WM contains axons with complex morphologies on an individual axon basis such as undulation, beading and non-circular cross sections, as well as non-trivial configurations including orientation dispersion and crossing bundles. On the other hand, the models used in simulation studies often represent axons in WM using simplistic geometrical representations such as parallel cylinders with uniform (Fieremans et al., 2010; Ford and Hackney, 1997; Nilsson et al., 2010, 2009) or polydisperse (Alexander et al., 2010; Hall and Alexander, 2009) radii. Some studies investigate the effect of differing configurations of fibres such as simple crossing (Rensonnet et al., 2017; Zhang et al., 2011a) and orientation-dispersed (Tariq et al., 2016; Zhang et al., 2012, 2011b) fibre bundles. A few groups generate WM numerical phantoms with complex fibre configurations for the application to tractography (Close et al., 2009; Neher et al., 2014); however realistic microstructural morphology is not the focus of these approaches. Other studies introduce more microstructural complexity into the numerical phantoms, typically only considering one mode of morphological variation at a time; some examples of this include harmonic beading (Budde and Frank, 2010; Landman et al., 2010), spines (Palombo et al., 2018b), undulation (Brabec et al., 2019; Nilsson et al., 2012) and myelination (Brusini et al., 2019).

Recently, a number of groups have attempted the challenge of combining these features to generate phantoms approaching the morphological complexity and density of real tissue. The most common approach to this is the packing of fibres into densely packed configurations (Close et al., 2009; Ginsburger et al., 2019, 2018; Rafael-Patino et al., 2018). The typical approach, as taken in the state-of-the-art MEDUSA algorithm (Ginsburger et al., 2019), is to generate a set of overlapping fibres decomposed into small segments and iteratively refine their positions to remove the overlap between them. Despite their recent progress, further advance of this class of techniques may be limited, because nature does not create fibres before attempting to pack them together. Instead, real axons are guided by chemical cues and fit into available space as they grow (Lowery and Vactor, 2009; Price et al., 2017). Mimicking the natural fibre genesis may prove important for building more realistic phantoms.

To this end, we propose Contextual Fibre Growth (ConFiG), an approach to generate WM numerical phantoms that emulates natural fibre growth. ConFiG generates WM numerical phantoms by growing fibres one-by-one, mimicking a set of key mechanisms which govern real axonal growth. A preliminary implementation of ConFiG was presented in (Callaghan et al., 2019). We assess the performance of ConFiG by measuring the impact of each of the biologically inspired mechanisms on the achievable phantom density and comparing against state-of-the-art MEDUSA phantoms. To test how realistic ConFiG phantoms are, we compare the microstructural properties of the phantoms to measured data from electron microscopy and compare simulated dMRI signal in the phantoms to real dMRI data.

The rest of the paper is organized as follows: Section 2 describes the ConFiG algorithm, Section 3 details the experiments outlined above and Sections 4, 5 summarise the contributions and discuss future work.

## 2 Methods

In this section we describe the ConFiG algorithm, beginning with an overview of the main components in the growth algorithm. We then describe the biological mechanisms motivating ConFiG and how each of these are implemented to give the final ConFiG algorithm.

### 2.1 Overview of the ConFiG algorithm

Given a set of morphological input parameters (target density, orientation distribution and diameter distribution), ConFiG generates a densely packed set of fibres by growing each fibre following a set of biologically motivated rules. The generation of ConFiG numerical phantoms happens in three main steps:

• STEP 1: Generate initial growth configuration from user inputs
• STEP 2: Grow the fibres using ConFiG growth algorithm
• STEP 3: Generate 3D meshes for dMRI simulation

Each of these steps are discussed in detail below.

**Fig. 1:**
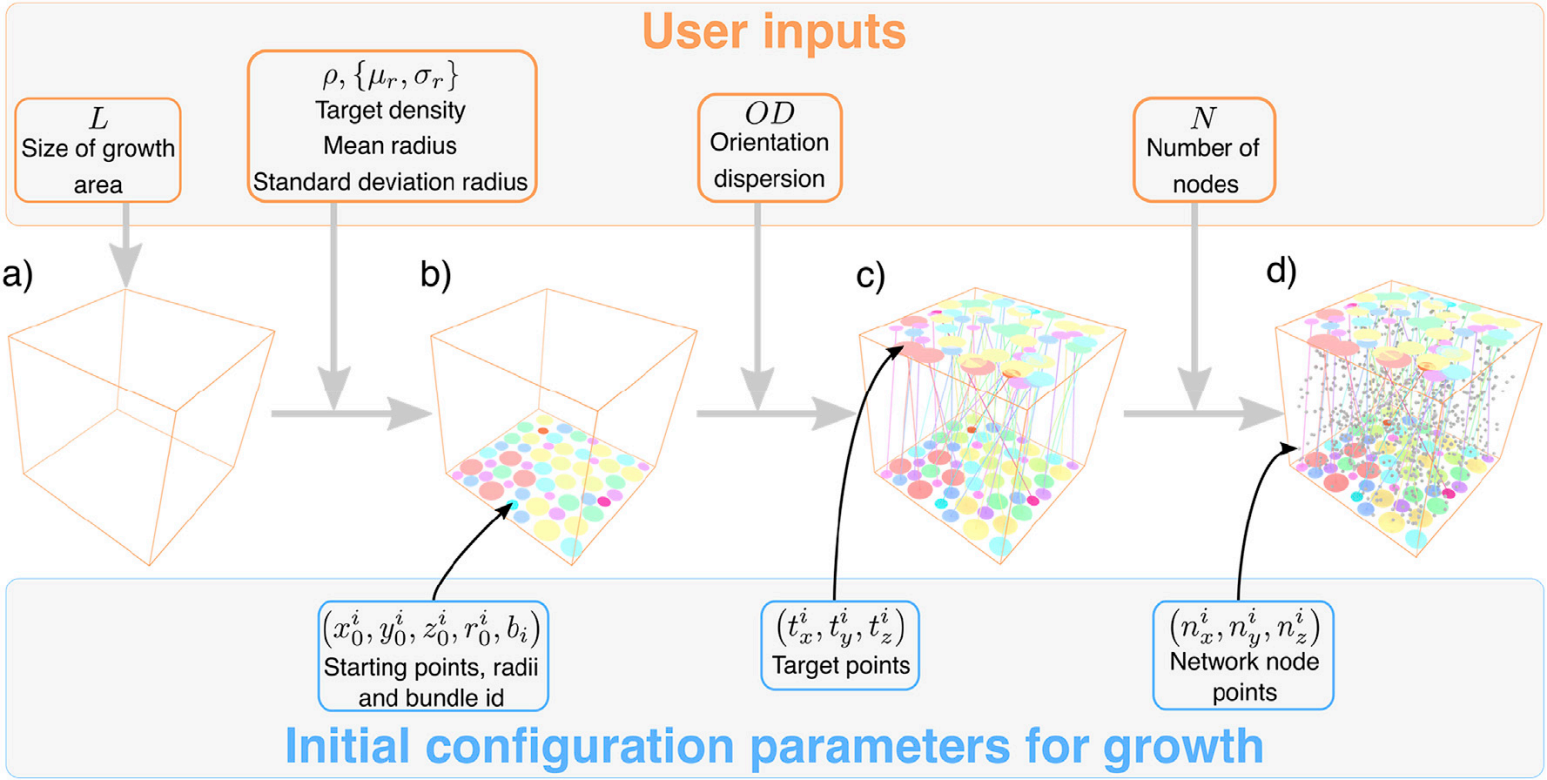
Inputs to the ConFiG algorithm for the single bundle case. L defines the size of the area of that the growth will take place in. The target density and fibre radius distribution govern the generation of starting points for each fibre by packing in 2D. Orientation dispersion parameters govern the generation of target points corresponding to each starting point. N defines the number of nodes to use when generating the network. In the case of multiple bundles, starting and target points are generated for each bundle and then combined into the same space which is filled with nodes for the network.

First, Step 1 is broken down in to three substeps as outlined in Fig. 1:

STEP 1.1: Generate fibre starting points (Fig. 1a–b). To generate a starting point for each fibre to grow from, ConFiG packs circles with the desired diameter distribution up to the target density (defined in terms of the desired fibre volume fraction) in 2D, following the approach taken in (Hall and Alexander, 2009).

STEP 1.2: Generate fibre target points (Fig. 1c). To encode the desired orientation distribution, each fibre has a direction drawn from the target distribution which gives a target point for the fibre to grow towards. As a demonstration of the flexibility of the framework, in this work we use the Watson distribution (Mardia and Jupp, 2008) for isotropic dispersion and the elliptically symmetric angular Gaussian distribution (Paine et al., 2018) for anisotropic orientation dispersion, however other orientation distributions can be defined according to the user’s needs.

STEP 1.3: Generate growth nodes (Fig. 1d). ConFiG uses a set of pseudorandomly placed points (nodes) to sample the space and encode which regions are occupied by existing fibres. This simplifies collision checking making growth more efficient than a direct collision detection approach involving growing each fibre one small step at a time and checking collisions with existing fibres (Callaghan et al., 2019).

**Fig. 2:**
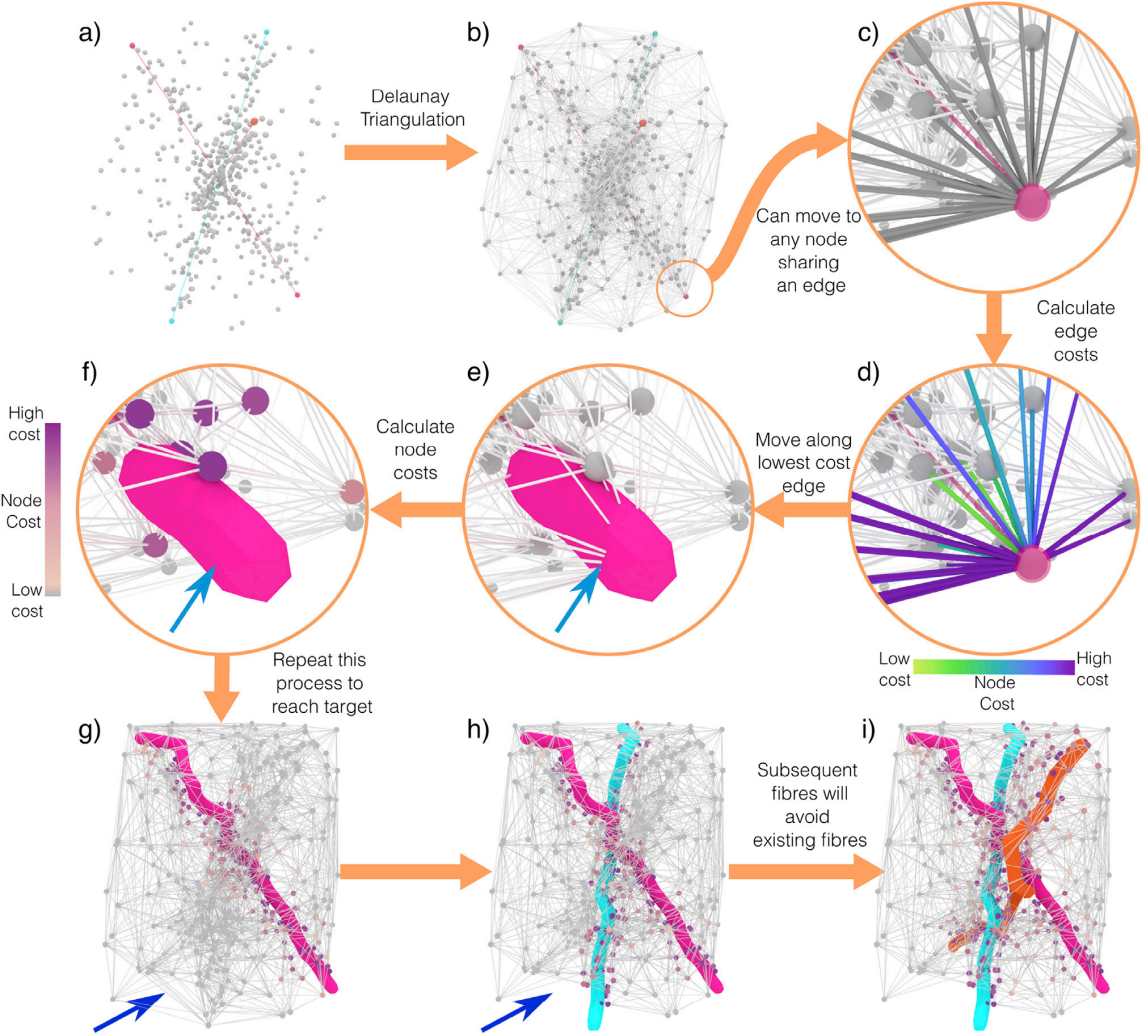
Overview of the basic growth algorithm in ConFiG. In this example, three fibres are shown with a growth network that only contains relevant nodes for the sake of visualisation. From the set of nodes, a network is constructed using the Delaunay triangulation. Each fibre then grows from node to node, along any edge connected to the current node. The node moved to will be the node with the lowest cost. Once a fibre segment has grown, the network nodes are updated to store information about which nodes are occupied or near to an existing fibres. This contributes to the cost function for any future fibres, penalising moving to nodes too close to existing fibres. It is not possible to move to any node now inside a fibre as indicated by the removal of this edges from the network (pairs of blue arrows show where this is happening). The next fibres grow, now avoiding existing fibres until all fibres have finished. See Supplementary Video 1 for an animation of this algorithm.

Second, Step 2, the main growth algorithm, is broken down into a series of substeps as outlined in Fig. 2:

STEP 2.1: Create growth network (Fig. 2a&b). In order to encode which nodes a fibre can move to from any other node, the growth nodes are connected using the Delaunay triangulation.

STEP 2.2: Grow one fibre step (Fig. 2 c-e). Fibres grow one-by-one in a random order along this network towards their target points while avoiding existing fibres. During growth, a fibre must choose in which direction it should grow. This direction is chosen in ConFiG by following a cost function motivated by biological axonal guidance mechanisms (Fig. 2d), described in Sections 2.3, 2.6.

STEP 2.3: Update the network (Fig. 2 f). The growth network is updated in order to store the information about the space this fibre is occupying so that future fibres can avoid it. The simplest way to do this is to store the minimum distance from each node in the network to any existing fibre as in (Callaghan et al., 2019). Additionally, another biologically motivated network updating strategy is described in Section 2.5.

STEP 2.4: Repeat steps 2.2 and 2.3 until fibre reaches target (Fig. 2g). By default in ConFiG, each fibre will grow completely before the next one starts, meaning that step 2.5 only needs to be performed once the fibre has finished growing. If fibres are allowed to grow concurrently, step 2.5 must be performed after each growth step.

STEP 2.5: Repeat steps 2.2–2.4 for remaining fibres (Fig. 2h–i). As noted in Fig. 2(e–h), as the network is updated, more and more nodes become inaccessible making the network sparser. This means that some fibres may reach a point from which they cannot grow any further and will become stuck. Biologically inspired mechanisms designed to address this point are described in Sections 2.4, 2.5.

Finally, Step 3, the meshing procedure, is briefly described below and in further detail in Section 2.8:

STEP 3: Generate 3D fibre meshes. After the growth process, each fibre will be represented by a series of connected 3D points and corresponding diameters at each point. In order to simulate diffusion MRI signals, these fibre skeleta need to be turned into 3D meshes. ConFiG uses a meshing procedure designed to eliminate overlap between fibres.

The basic ConFiG growth algorithm described here is illustrated in Fig. 2, with an animation of the algorithm in Supplementary Video 1. The remainder of this section outlines the biological process governing real axonal growth, and how these processes motivated the final implementation of the ConFiG algorithm.

Supplementary video related to this article can be found at https://vimeo.com/433661018 or https://doi.org/10.1016/j.neuroimage.2020.117107

The following is the supplementary data related to this article:

Video 1 Illustration of the ConFiG growth algorithm overviewed in Section 2.1 and Fig. 1, Fig. 2. Starting and target points for each fibre are generated before the growth network is created and the fibres grow along it following a biologically motivated cost function. See also https://vimeo.com/433661018.

### 2.2 Biological motivation for ConFiG

In nature, axons grow following chemical cues in their environment through various mechanisms which either attract or repel fibres to guide their growth (Dent et al., 2011; Lowery and Vactor, 2009; Mortimer et al., 2008; Polleux and Snider, 2010; Price et al., 2017; Rauch et al., 2013; Sakisaka and Takai, 2005). In an attempt to emulate real axonal growth, mechanisms motivated by the following guidance processes have been integrated into ConFiG:

• Chemoattraction – the process by which fibres are attracted to diffusible chemical cues in their environment (Mortimer et al., 2008; Price et al., 2017).
• Fibre collapse – a response to a chemorepulsive source whereby a fibre withdraws and regrows in a different direction (Rauch et al., 2013).
• Cell adhesion molecules – chemical signals on the surface of cells which guide axons that come into contact with them (Sakisaka and Takai, 2005).
• Fasciculation – the process by which multiple axons come together to form bundles (Price et al., 2017; Šmít et al., 2017).

**Fig. 3:**
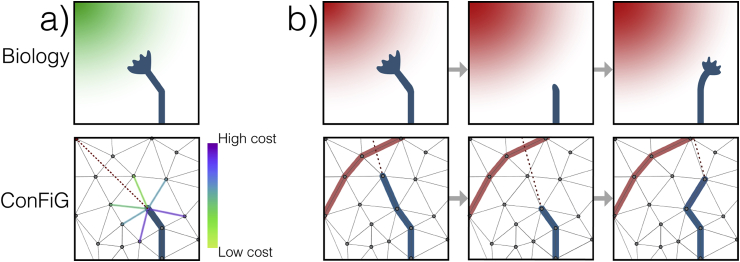
Illustration of two of the biological motivations and how they are implemented in ConFiG. a) Growth towards the target is enforced by means of a cost function encouraging growth towards the target point. b) Fibre collapse is implemented by allowing the fibre to move backwards if it reaches a node from which there are no viable steps. The biological figures are adapted from (Price et al., 2017).

**Fig. 4:**
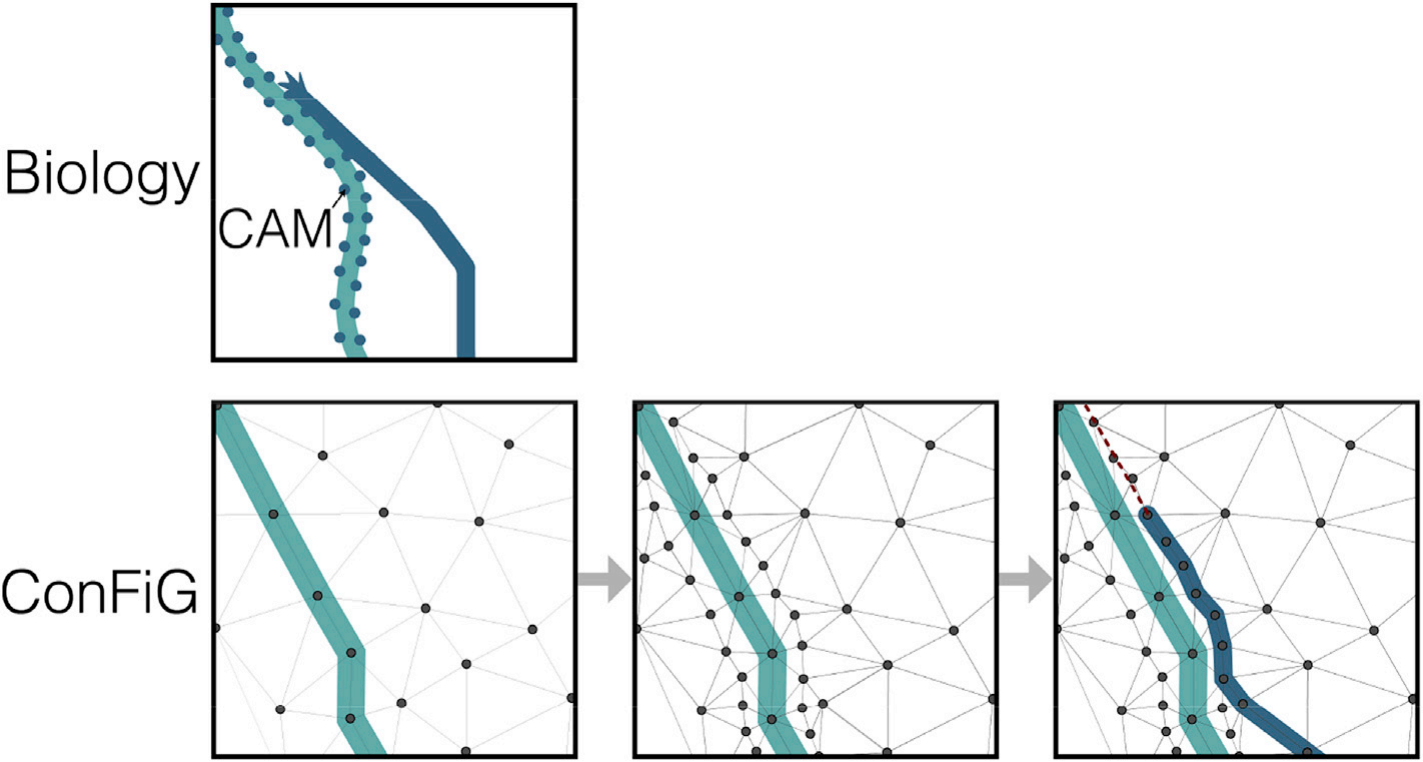
Illustration of the contact guidance axonal growth mechanism and the dynamic growth network implemented in ConFiG. The dynamic growth network is implemented as a set of points added around each fibre after growth, enable future fibres to more easily grow along/around existing fibres.

**Fig. 5:**
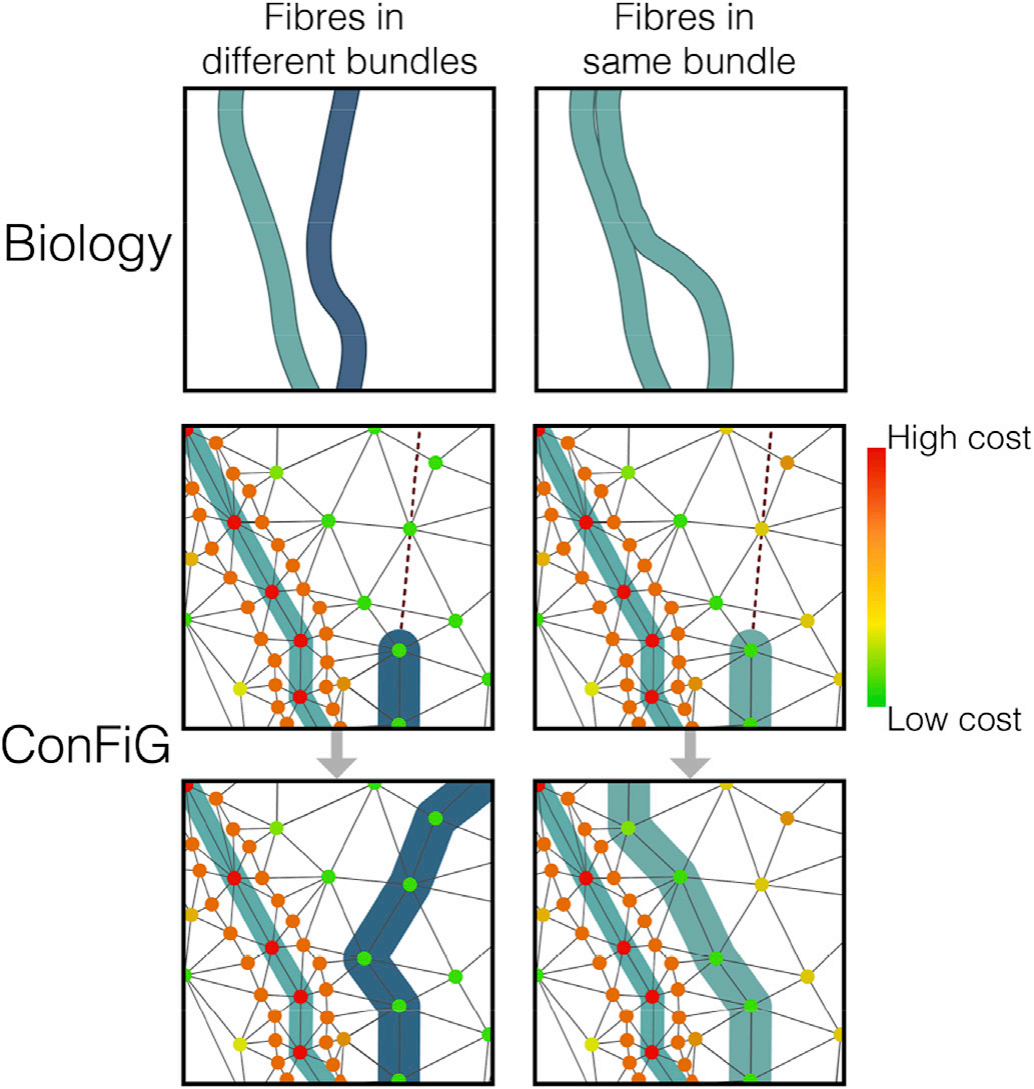
Illustration of how the labelled pathway hypothesis is expected to work in biology and its ConFiG counterpart. Fasciculation is implemented using the cost function term in Eq. (1) which means that fibres in the same bundle are encouraged to stay close to one another.

The following sections detail how mechanisms motivated by these biological processes are implemented in ConFiG while Fig. 3, Fig. 4, Fig. 5 illustrate these biological processes alongside their ConFiG counterparts.

### 2.3 Chemoattraction

As mentioned in Section 2.1, as a fibre grows it must choose in which direction it will move. One of the main processes governing the guidance of real axons is chemotropism; a process by which axons respond to diffusible chemical cues in their environment. One key chemotropic mechanism is chemoattraction, in which fibres are attracted along a chemical gradient towards a target region (Price et al., 2017).

To approximate this chemoattractive mechanism, each fibre is encouraged to grow towards its target point (i.e. the target point acts like a chemoattractive source). From any node in the growth network, the fibre will move along an edge that takes it towards its target while avoiding existing fibres according to a cost function (Callaghan et al., 2019). The chemoattractive mechanism and its ConFiG counterpart are illustrated in Fig. 3a.

From a starting node, s, the candidate nodes, c, that the fibre can move to are any nodes that share an edge with s. In addition to its position, each network node stores the maximum fibre diameter, $d_{c}$, that can be sustained at that node without intersecting another fibre. The fibre will move to a candidate node according to a cost function consisting of two terms; $l_{t},$ which penalises taking very large steps or moving away from the target point, t, and $l_{d},$ which penalises moving to a position where $d_{c}$ is low meaning that the fibre will have to shrink. The cost function for a fibre at a position, s, to move to a candidate node, c, given a target point, t, is (Callaghan et al., 2019)

(1) $$l=\;l_{t}+fl_{d}\;,$$

where

(2) $$l_{t}=\;\frac{1}{2}\cdot \frac{\left|s-c\right|}{1+\;\left|s-c\right|}\cdot \left(1-\;\frac{\left(c-s\right)\cdot \left(t-s\right)}{\left|c-s\right|\left|t-s\right|}\right)\;,$$

(3) $$l_{d}=\max\left(0,\;\frac{1}{d_{0}}\left(d_{0}-d_{c}\right)\right).$$

Here, $d_{0}$ is the target diameter of the fibre and f is a weighting factor between the two terms. In this work, f is fixed to 0.2 to more strongly weight growth towards the target.

The next node for a fibre will be the candidate node which has the lowest cost according to Equation (1). This method of finding a path through the triangulation by choosing the lowest cost node at each position amounts to a greedy best-first pathfinding approach with a heuristic given by Equation (1).

Growing fibres along the network using just this chemoattractive mechanism is the minimal implementation of ConFiG that will generate substrates to try and meet the morphological inputs. There are some limitations to this minimal approach however; the greedy growth and the sparse sampling of the space means that fibres can grow into regions from which they cannot grow further and become stuck. Additionally, in this approach, fibres grow independently of one another, whereas real fibres grow forming bundles in the process known as fasciculation.

Sections 2.4-2.7 describe further mechanisms which were added to enable ConFiG to address these limitations in order to meet more complex morphological priors (e.g. high density and orientation dispersion together).

### 2.4 Fibre collapse

As mentioned in Section 2.3, in ConFiG a fibre can become stuck when there are no possible next steps because all neighbouring nodes are inaccessible. In an attempt to ameliorate this a process mimicking fibre collapse was implemented, illustrated in Fig. 3b.

In ConFiG fibre collapse, the fibre will move back by an initial distance, $g_{0}$, and regrow from there avoiding any nodes in the route it took previously. If the fibre becomes stuck again, it will move back by a further distance, $g_{0}+\;\delta$, where δ is the additional distance to step back. This process is repeated until the fibre reaches the target or gets stuck a user-defined maximum number of times. In this work, $g_{0}=2\;\mu m$ and δ=5μm in an approximation of the biological fibre collapse process investigated by Rauch et al. (2013) who show fibres collapsing up to 25 μm back towards the soma. The maximum number of steps back is set to 5, meaning that the maximum step back is 27 μm, in line with real fibres. If there is no possible route after 5 attempts then the fibre will stop growing and will be removed from the phantom. This process of removing stuck fibres means that the resulting substrate may not always have the same density as the input desired fibre density.

### 2.5 Dynamic growth network

In the preliminary implementation of ConFiG (Callaghan et al., 2019), the network nodes were initialised pseudorandomly within the growth region and once initialised, the growth network was static, meaning that the nodes and edges of the network were fixed. This limited the growth to the specific instantiation of the network and it could not adapt to where fibres were once they had grown. Furthermore, as illustrated in Fig. 2, as fibres grow, many nodes become inaccessible due to being within fibres meaning that the network becomes gradually sparser.

A dynamic growth network was implemented to ameliorate these effects. Now, once a fibre has reached the target, a number of nodes, $N_{added}$, are generated around the path of the fibre. This gives a denser sampling of the space in regions in which fibres exist and serves to give subsequent fibres more nodes to use to grow along or around that fibre, helping to increase the achievable density by limiting the number of fibres which get stuck. In this work, where the dynamic network is used, $N_{added}=2500$.

This is also loosely motivated by the contact guidance mechanism in which axons are attracted to or repelled by chemical cues on the surface of other cells, known as cell adhesion molecules (CAMs). Here, the added points act like CAMs meaning that a future fibre which grows can use these points near to the fibre to grow around or along it as if it were following contact guidance cues. Fig. 4 shows how CAMs work in biological axonal growth alongside the ConFiG dynamic network, illustrating the parallels between the two.

### 2.6 Axon fasciculation

One particular role CAMs play is in axon fasciculation, the process in which axons follow a so-called pioneer axon closely, forming a bundle (Price et al., 2017; Sakisaka and Takai, 2005). To mimic the process of axon fasciculation, the term in the cost function penalising moving into regions in which the fibre had to shrink, $l_{d}$ (Equation (3)), was altered to be conditional on which fibre bundle is closest.

A fibre, f, with a target diameter, $d_{0}$, moving to a candidate node, c, which has a maximum sustainable diameter $d_{c}$ will now have $l_{d}$ given by:

(4) $$l_{d}=\;\left\{ \begin{array}{l} \max\left(0,\;\frac{1}{d_{0}}\left(d_{0}-\;d_{c}\right)\right)\;\;\;\;\;\;if\;b_{c}\neq \;b_{f}\\ \mathrm{abs}\left(\frac{1}{d_{0}}\left(d_{0}-\;d_{c}\right)\right)\;\;\;\;\;\;\;\;\;\;\;\;if\;b_{c}=\;b_{f} \end{array}\;,\right.$$

Where $b_{f}$ is an index identifying the bundle that fibre f belongs to and $b_{c}$ is the index of the bundle that is closest to c (i.e. the index of the bundle of the fibre that set $d_{c}$). This means that when c is closest to the same bundle as f, the cost function penalises moving away from that bundle as well as shrinkage, whereas when the bundles differ, it only penalises shrinkage.

This new form of the cost function encourages fibres of the same bundle to stick together while still avoiding fibres of different bundles, inspired by the labelled pathway hypothesis, which states that axons join different fascicles based on different CAMs expressed on the fibres (Price et al., 2017). In this case, bundle indices $b_{c}$ and $b_{f}$ act like different identifying CAMs. Fig. 5 shows how this fasciculation process is expected to happen in biology alongside how the improved cost function encourages a similar process in ConFiG.

### 2.7 Global optimisation

Since the growth of fibres in ConFiG takes place on a discrete network of points, the final positions of fibre nodes may be suboptimal for achieving the maximum density. In other words, certain fibres’ nodes may be closer to other fibres than they would ideally be in order to reach their target diameter (i.e. the fibre has had to shrink its diameter at that node).

To mitigate against this, a global optimisation step was added at the end of the growth in a procedure similar to MEDUSA (Ginsburger et al., 2019). For each point, i, that is part of a fibre, its nearest n neighbours (j∈NN(i)) from other fibres are found; in this work n=10. The distance to all of the neighbours is found and the point’s position is updated from these distances according to the update vector, $\vec{u_{i}}$

$$\vec{u_{i}}=\;\sum_{j\;\in \;NN\left(i\right)}D\left(i,j\right)\cdot \left(\;\vec{p_{i}}-\;\vec{p_{j}}\;\right)\;,$$

where $\vec{p_{i}}$ and $\vec{p_{j}}$ are the locations of point i and j. D(i,j) is the function determining whether the interaction is repulsive or attractive:

$$D\left(i,\;j\right)=\mathrm{sgn}\left(r_{i}+r_{j}-\left|\vec{p_{i}}-\;\vec{p_{j}}\right|\;\right).$$

Here, sgn is the signum function and $r_{i}$ and $r_{j}$ are the target radii of point i and j. The sum of these radii is the desired distance between the points since that means the fibres are just touching. D(i,j) imposes that the force is repulsive if the points are closer together than the desired radius and attractive if they are further apart. The update vector is scaled such that if $\left|\vec{u_{i}}\right|\to >0.2r_{i}$, the update vector is rescaled so that $\left|\vec{u_{i}}\right|=0.2r_{i}$. This acts to prevent the update vector from becoming very large.

There is some biological evidence that this kind of interaction between fibres is important in the fasciculation process. The fasciculation process described in Section 2.4 relies on CAMs detected at the tip of a growing axon, however some studies provide evidence for fasciculation through interactions along axon shafts, known as zippering (Barry et al., 2010; Šmít et al., 2017; Voyiadjis et al., 2011). In zippering, nearby axon shafts attract one another to form more closely packed fascicles, which is a similar process to the global optimisation process in ConFiG.

### 2.8 Creation of 3D meshes

As mentioned in Section 2.1, following the growth process ConFiG fibres will be represented by a series of connected points and corresponding radii. To convert these skeleta into 3D meshes, the ConFiG meshing procedure uses Blender (https://blender.org) and is built on the SWC mesher addon (https://github.com/mcellteam/swc_mesher).

**Fig. 6:**
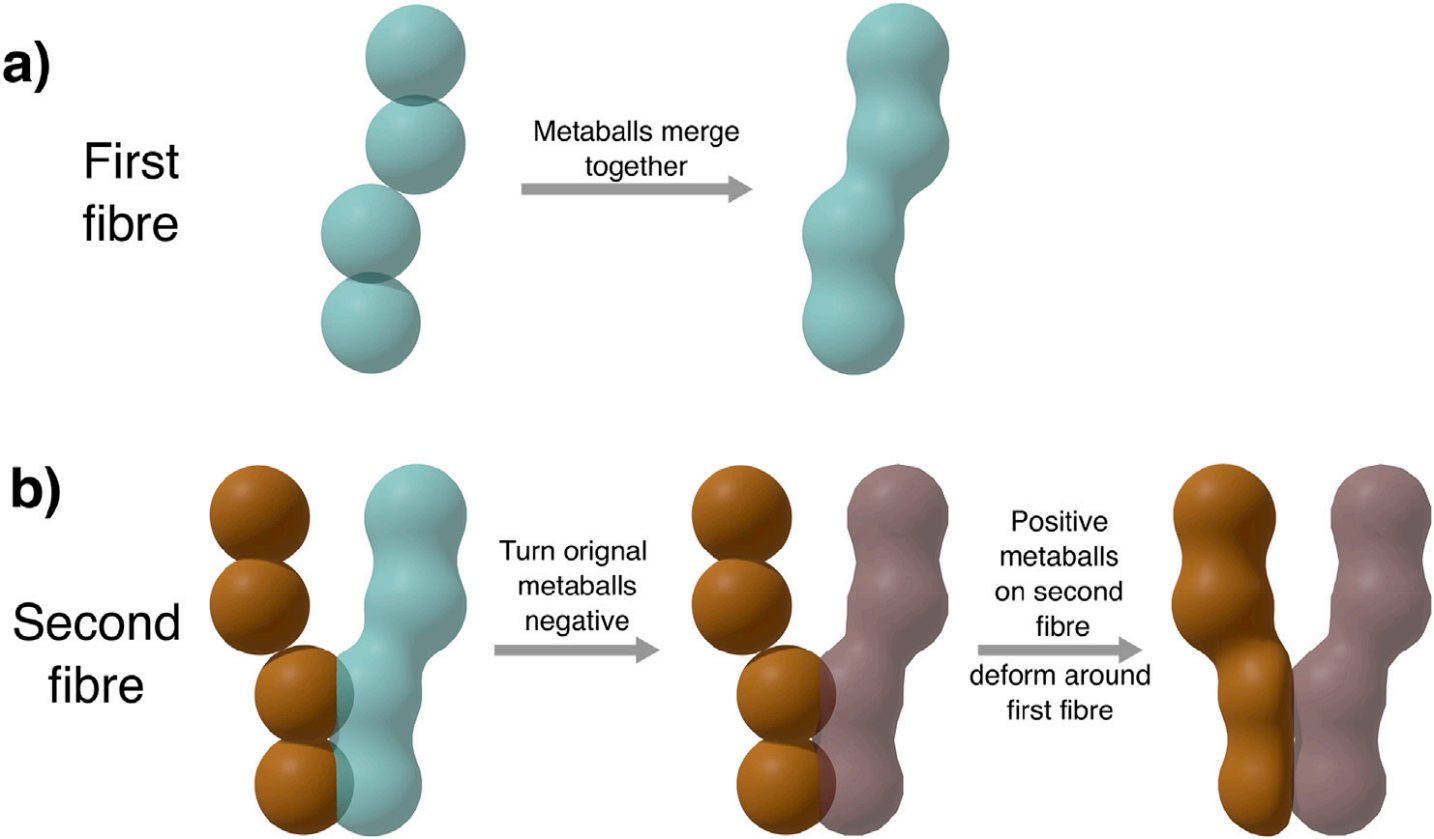
Demonstration of the meshing procedure in ConFiG. The first fibre is created using metaballs to create a smooth surface. The second, and following fibres will be created using negative metaballs for any fibres that intersect in order to deform around them. Note that in practice, more spheres will be much more closely placed along the skeleton to create a smoother surface.

ConFiG meshes are constructed using Blender metaballs, an implicit surface representation which is the isosurface of a function; typically a function analogous to the electric potential from a point charge. When two metaballs come close to one another, the fields combine and the surfaces will merge to form a smooth surface. By placing a series of metaballs along the skeleton of each fibre, a smooth surface is formed for each fibre one-by-one as shown in Fig. 6a. Supplementary Fig. 1 demonstrates that the ConFiG meshing procedure does not impact the diffusion dynamics compared to a straight cylinder.

When fibres are densely packed, the surfaces from neighbouring fibres may overlap. To account for this, a meshing procedure was developed in which fibres can deform around nearby fibres to avoid overlap. The metaball surface for one fibre is created as described above. This surface is then turned into a triangulated mesh, however the metaballs are retained. The metaball potential is then turned negative, meaning that rather than merging with any future nearby metaball surfaces, it will repel them, as shown in Fig. 6b. This means that subsequent fibres which are meshed very close to, or overlapping with, existing fibres will deform organically to resolve the intersection, thus creating a series of completely non-intersecting fibre meshes which can be used by the dMRI simulator.

### 2.9 Summary of ConFiG input parameters

Table 1 summarises the key parameters that govern the generation of ConFiG phantoms. Parameters are split into those which define the target microstructural morphology and those which define the instantiation of the growth algorithm. For each parameter, the theoretical range is reported alongside the practical range that has been tested so far. This is due to stochastic nature of the algorithm and the interdependence of the parameters. For instance a very large substrate is possible if very large fibres are chosen, but likely impossible with very small fibres since this will require a very large number of fibres and run into memory limitations.

**Table 1:** Summary of ConFiG parameters split into parameters which define the target microstructural morphology and parameters which define the instantiation of the growth algorithm. For each parameter the theoretical range is reported as well as the practical range that has been tested so far.

Parameter		Meaning		Theoretical Range	Practical limits tested
*Target microstructure parameters*	L	Size of growth region		$R_{+}^{3}$	[0,0,0] -> [50,50,50] μm
	ρ	Fibre volume fraction		[0,1]	[0,0.8]
	$\mu_{\text{r}}$	Mean radius		$R_{+}$	[0.5, 2] μm
	$\sigma_{\text{r}}$	Standard deviation radius		$R_{+}$	[0.1, 0.5] μm
	GAD	Global angular dispersion	Watson: κ	$R_{+}$	[4, 100]
			ESAG $\left\{ \begin{array}{l} \mu \\ \gamma \end{array}\right.$	$R_{+}$	[2, 10]
				$R_{+}^{2}$	[2, 2] -> [10, 10]
*Growth algorithm parameters*	N	Number of growth nodes		N	[0,10^7^]
	f	Cost function weighting term		[0,1]	[0,0.5]
	${\text{g}}_{0},\;\delta$	Fibre collapse initial and subsequent step length		$R_{+}$	[1, 5], [1, 5] μm
	${\text{N}}_{\text{added}}$	Dynamic network no. nodes added		N	[0, 5000]

## 3 Experiments

In order to assess the performance of ConFiG, a range of experiments were performed. The first set of experiments were performed in order to explore the impact of each of the biologically inspired growth mechanisms. Another set of experiments aimed to show that ConFiG is able to generate substrates with realistic microstructure by comparing generated substrates with real tissue. Additionally, the relationship between the user-specified target morphology and the final output morphology was investigated by comparing resulting phantoms to their inputs (target density and orientation distribution). Finally, a simulation experiment was performed to assess how well ConFiG phantoms can be used to generate realistic diffusion MRI data. The rest of this section outlines these experiments.

### 3.1 Testing the performance of ConFiG

In order to test how each of the biological mechanisms proposed in Section 2 impacted on the resulting phantoms, an experiment was devised to measure how phantoms changed when each mechanism was introduced. Four scenarios of interest were generated using several variants of the ConFiG algorithm that included these mechanisms either one at a time or all at once, attempting to grow phantoms as densely as possible:

• one bundle of parallel fibres, target density 75%
• one bundle with Watson distributed fibres (κ = 8), target density 75%
• two perpendicular crossing bundles, intra-bundle target density 40%
• three mutually perpendicular crossing bundles, intra-bundle target density 30%

These target densities were chosen to ensure that the centre of the phantom (i.e. the crossing region for crossed bundles) had a high target density whilst ensuring that each bundle had a reasonable number of fibres to begin with (>50).

The ConFiG variants were tested by generating phantoms for each of the scenarios starting with the same initial conditions. Each phantom was generated 5 times with a different random seed and results averaged across the seeds.

To investigate the impact of the biological mechanisms on dMRI simulation, a comparison was made between real dMRI signals and simulations from ConFiG phantoms. The NODDI model (Zhang et al., 2012) was fitted to a WM ROI in the corpus callosum of a Human Connectome Project (HCP) (Van Essen et al., 2012) subject to provide sensible input parameters (target fibre density and orientation dispersion) for ConFiG to generate phantoms. We generated phantoms using the two extreme cases: the minimal growth case only using chemoattraction, and the complete ConFiG algorithm using all mechanisms. Whilst the random nature of ConFiG means that the resulting phantom will not have morphology exactly matching the input parameters, this approach ensured that the phantoms were reasonable for this proof of concept experiment.

The dMRI signal was simulated in the phantoms using Camino (Cook et al., 2006; Hall and Alexander, 2009) with identical simulation conditions in both cases and the measurement scheme corresponding to the HCP dMRI sequence (Sotiropoulos et al., 2013a). An important consideration when performing dMRI simulations is the size of the substrate relative to the diffusion length. The phantom should be large enough that it is bigger than the diffusion length, but not so large as to require excessive computational resources. Owing to the relatively long diffusion time (43 ​ms) in the HCP sequence, phantoms were extended with reflected copies (Fieremans and Lee, 2018; Lee et al., 2020) to increase their effective size relative to the diffusion length scale.

All dMRI simulations in this work used a bulk diffusivity $D=2.0\mu m^{2}/ms$ in agreement with values used in similar Monte Carlo simulations (Hall and Alexander, 2009; Nilsson et al., 2009; Rensonnet et al., 2017) with $10^{5}$ spins and 2000 timesteps. Standard Camino periodic boundaries were used (Hall and Alexander, 2009), with dMRI signal was generated from a central region 75% the size of the total phantom to avoid boundary effects (Panagiotaki et al., 2010).

### 3.2 Microstructural measures

In order to test how realistic the microstructure generated using ConFiG is, microstructural measurements of diameter distribution and orientation distribution were calculated using methods to be comparable with previous studies on ex-vivo tissue (Abdollahzadeh et al., 2019; Lee et al., 2019).

**Fig. 7:**
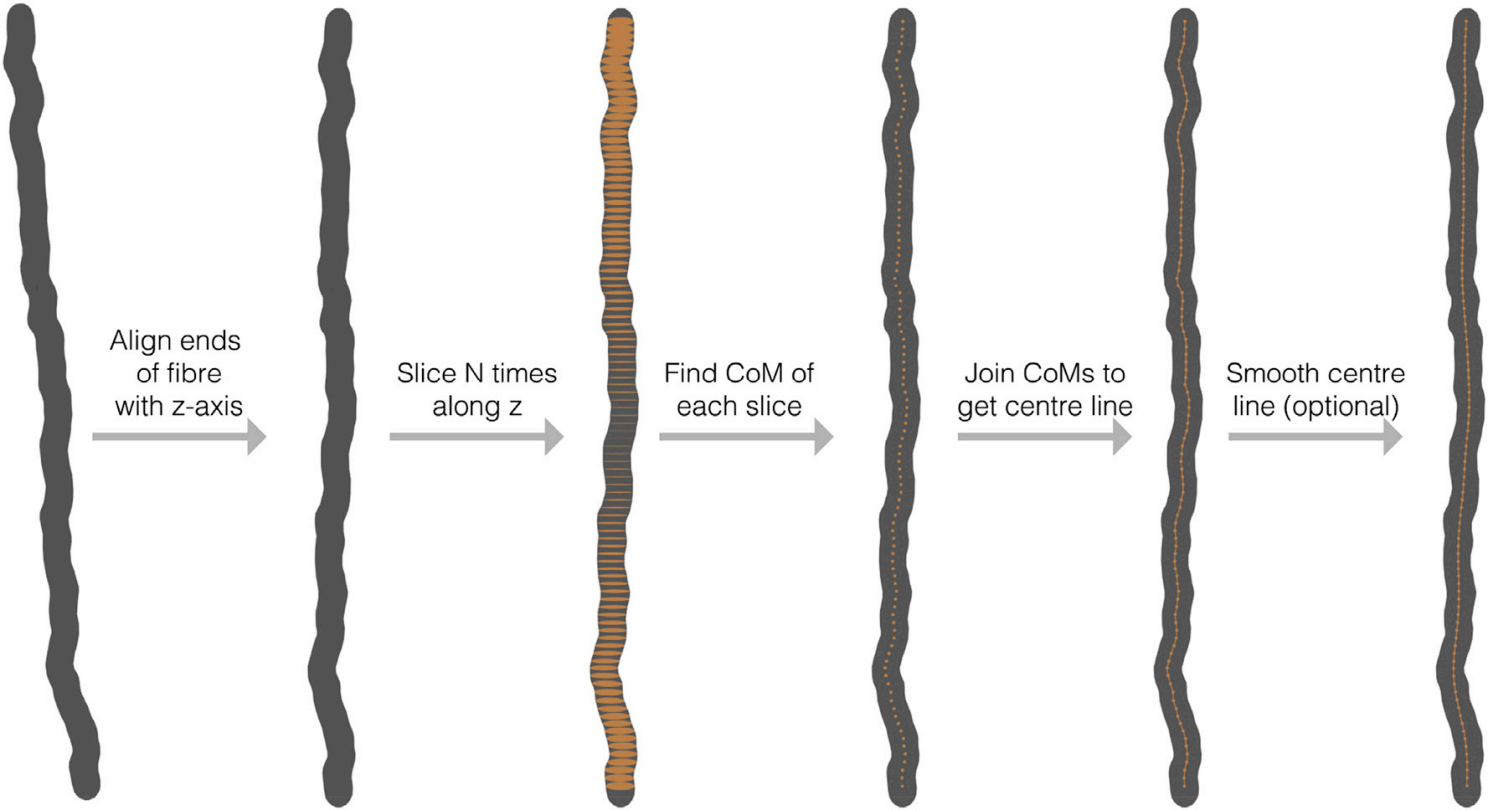
Centre line extraction of fibres. Each fibre was sliced N times along the z-axis, connecting the centre of mass of each slice to create the points in the centre line. This line could then be optionally smoothed according the diffusion time coarse graining effect, as in (Lee et al., 2019).

A centre line is generated from each of the fibre meshes by aligning the ends of each fibre with the z-axis and connecting the centre of mass of 100 equidistant slices through each fibre, following the approach taken by Lee et al. (2019). This is illustrated in Fig. 7.

**Fig. 8:**
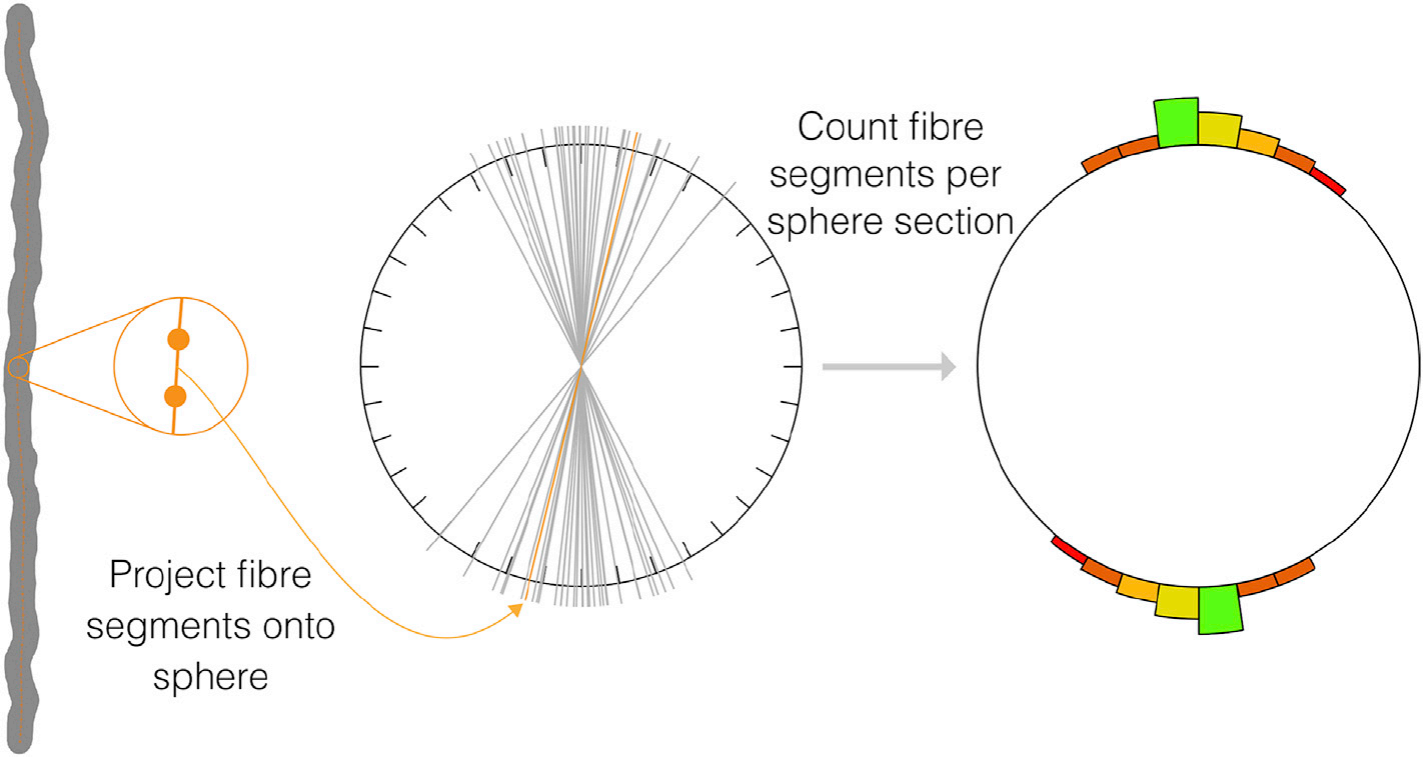
Orientation distribution calculation. Each segment of a fibre was projected onto the surface of a triangulated sphere, here illustrated with a sectioned circle. For each section in the sphere, the number of fibre segments going through that section was used to colour and/or raise the surface to visualise the orientation distribution. Since the diffusion process is symmetric about the origin, each fibre segment was projected onto the sphere forwards and backwards.

Each segment in this centre line could then be used to assess the microstructure of the phantom. The direction of each segment was used to assess the orientation distribution of the phantom, illustrated in Fig. 8. Following the approach of Lee et al. (2019), the direction of each segment was projected onto the surface of a triangulated unit sphere (Womersley, 2018). For each triangle, the number of segments pointing in that direction was used to colour the triangle to visualise the orientation distribution.

A second approach was devised to better visualise orientation distributions in 3D to aid differentiation of crossing bundles and antipodal symmetry. In this approach each vertex was raised above the surface of the sphere proportionally to the number of segments pointing in its direction as illustrated in Fig. 8.

**Fig. 9:**
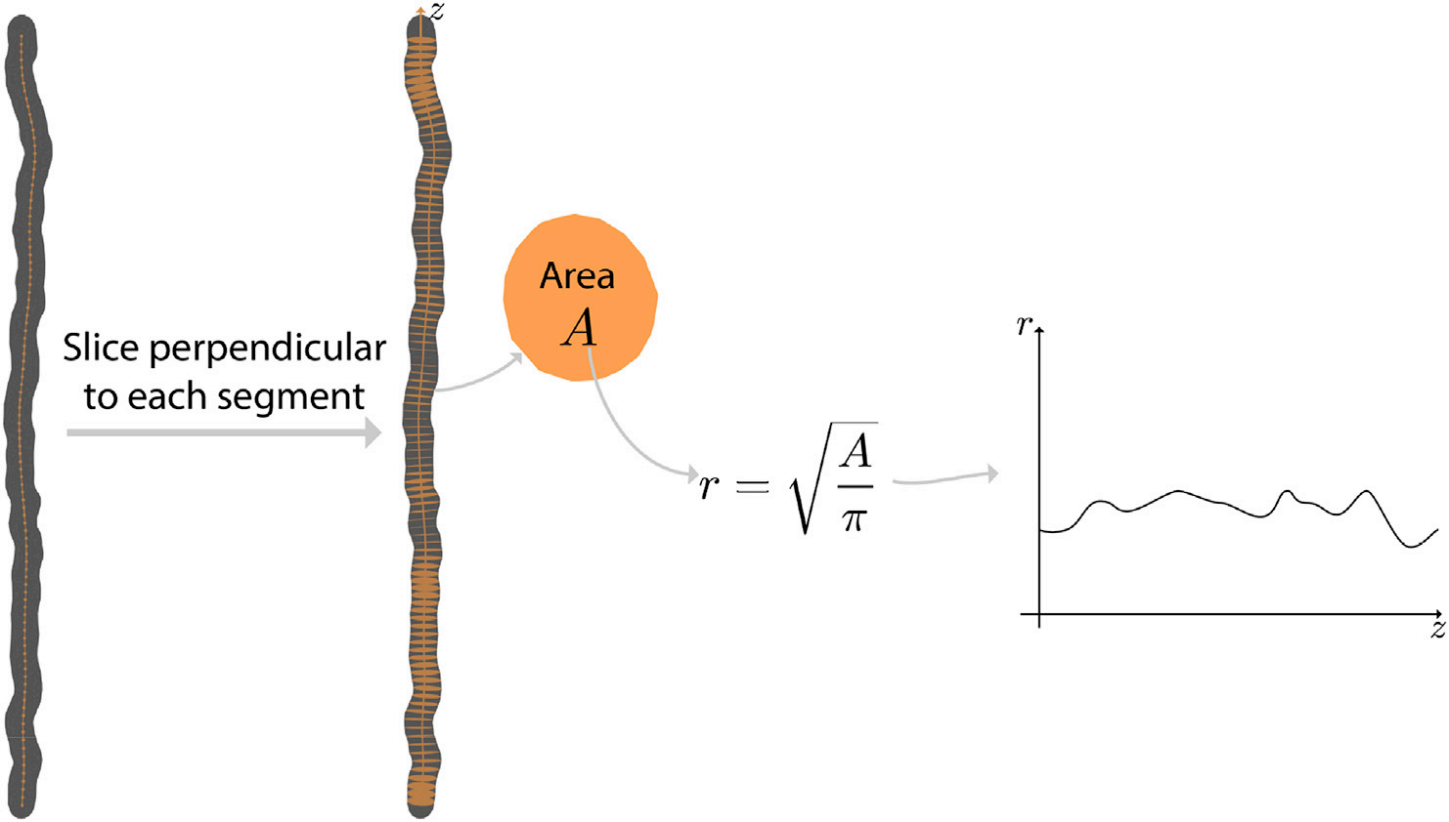
Calculation of the diameter distribution. A slice is taken through each fibre perpendicular to every segment in the centre line. The area of each of these slices is used to find a circle equivalent radius or diameter using $A\;=\;\pi r^{2}$.

To measure the diameter profile along fibres, the direction of each segment gave the normal to a plane used to cut the fibre using Boolean intersection to give a cross section of each fibre at each segment. The diameter profile along the axon was generated by calculating the equivalent diameter of a circle with the same area as the fibre cross section. This process is illustrated in Fig. 9.

### 3.3 Virtual histology

Virtual histological slices were generated to compare ConFiG substrates to real white matter analysed using histology. Histological slices were found by calculating the Boolean intersection of a cutting plane with the generated fibre meshes using Blender (https://blender.org). A myelin sheath was added to the fibres when generating virtual histology for visualisation purposes. Virtual histological images were rendered with a resolution of 5 ​nm ​× ​5 ​nm x 100 ​nm, chosen to be comparable to real histological white matter measurements (Abdollahzadeh et al., 2019; Lee et al., 2019).

In order to compare ConFiG virtual histology to real histology, virtual histological slices were rendered in binary black and white to compare against intra-axonal segmentations from (Lee et al., 2019). Slices from real histology, ConFiG phantoms and a parallel cylinder phantom were processed using the MorphoLibJ plugin for ImageJ (Legland et al., 2016; Rueden et al., 2017; Schindelin et al., 2012; Schneider et al., 2012) to extract morphological features: circularity ($4\pi \;\times \;Area/Perimeter^{2}$), convexity (Areaofshape/Areaofconvexhull), eccentricity of fitted ellipse and $Area/\pi r_{max}^{2}$ for each axon. Axons touching the edge of the image were removed since truncation from the image edge would skew these microstructural metrics.

### 3.4 Relationship between input and output morphology

As mentioned in Section 2, the nature of the ConFiG growth algorithm means that the microstructural morphology of the phantoms may not match the user input. Some fibres may become stuck and fibres cannot typically grow in a straight line, affecting the density and orientation distribution.

To investigate this, we generated a series of ConFiG phantoms with Watson distributed orientation dispersion with κ=[8,10,15,20,30,50,100] and target density, ρ ​= ​75%. The target density of 75% is chosen as this is the upper limit of what is achievable empirically and towards the higher end of expected axonal volume fraction. Additionally, with 75% achieved, lower densities can be generated easily, either by running ConFiG in full, or simply by removing or shrinking fibres.

The mean and standard deviation of the angle from z, $\mu_{\theta }$ and $\sigma_{\theta }$ respectively, for each κ was calculated by taking 10000 samples from the Watson distribution and this was compared to $\mu_{\theta }$ and $\sigma_{\theta }$ of the ConFiG fibres. Additionally, the density of the ConFiG phantoms was compared to the target density of 75%. Each phantom was generated in a 20 ​× ​20 ​× ​20 μm region, using $2.5\times 10^{6}$ nodes in the growth network.

### 3.5 Diffusion MRI simulation

To qualitatively verify that the simulated diffusion MRI signals from ConFiG phantoms are realistic, simulated signals from ConFiG phantoms were compared to real HCP data (Sotiropoulos et al., 2013b; Van Essen et al., 2012).

In the real data, the fibre orientation distribution (FOD) was fit in each voxel using constrained spherical deconvolution in MRTrix (Tournier et al., 2019, 2007). Voxels were selected in regions of interest in the midbody of the corpus callosum (CC), internal capsule (IC), regions in which a single bundle of fibres is found from the FOD. A third voxel was selected in which three crossing fibre populations were found from visual inspection of the FOD (TC).

In each voxel, the diffusion tensor was fit to the signal and the principal eigenvector used to define a major direction of diffusion in the voxel, n. From this, the normalised diffusion weighted signal was plotted against |n⋅G|, where G is the gradient direction. Additionally, the direction averaged signal was calculated for each b-shell.

To attempt to generate representative microstructure for each voxel using ConFiG, the NODDI model (Zhang et al., 2012) was fitted to the signal to give some initial parameters for ConFiG. Most importantly, the value of κ for the Watson distribution (Mardia and Jupp, 2008) estimated using NODDI was used to initialise the orientation dispersion in the ConFiG phantoms used to represent CC (κ=6.2) and IC (κ=5.5) regions. To represent the TC voxel, a phantom generated using three mutually perpendicular crossing bundles was used.

ConFiG phantoms were grown using these initial conditions and the diffusion MRI signal simulated using the Camino Monte Carlo diffusion MRI simulator (Hall and Alexander, 2009). For each phantom, the same processing as with the real data was performed, finding the direction dependent and direction averaged signal per b-shell.

## 4 Results

### 4.1 Impact of biological mechanisms

**Fig. 10:**
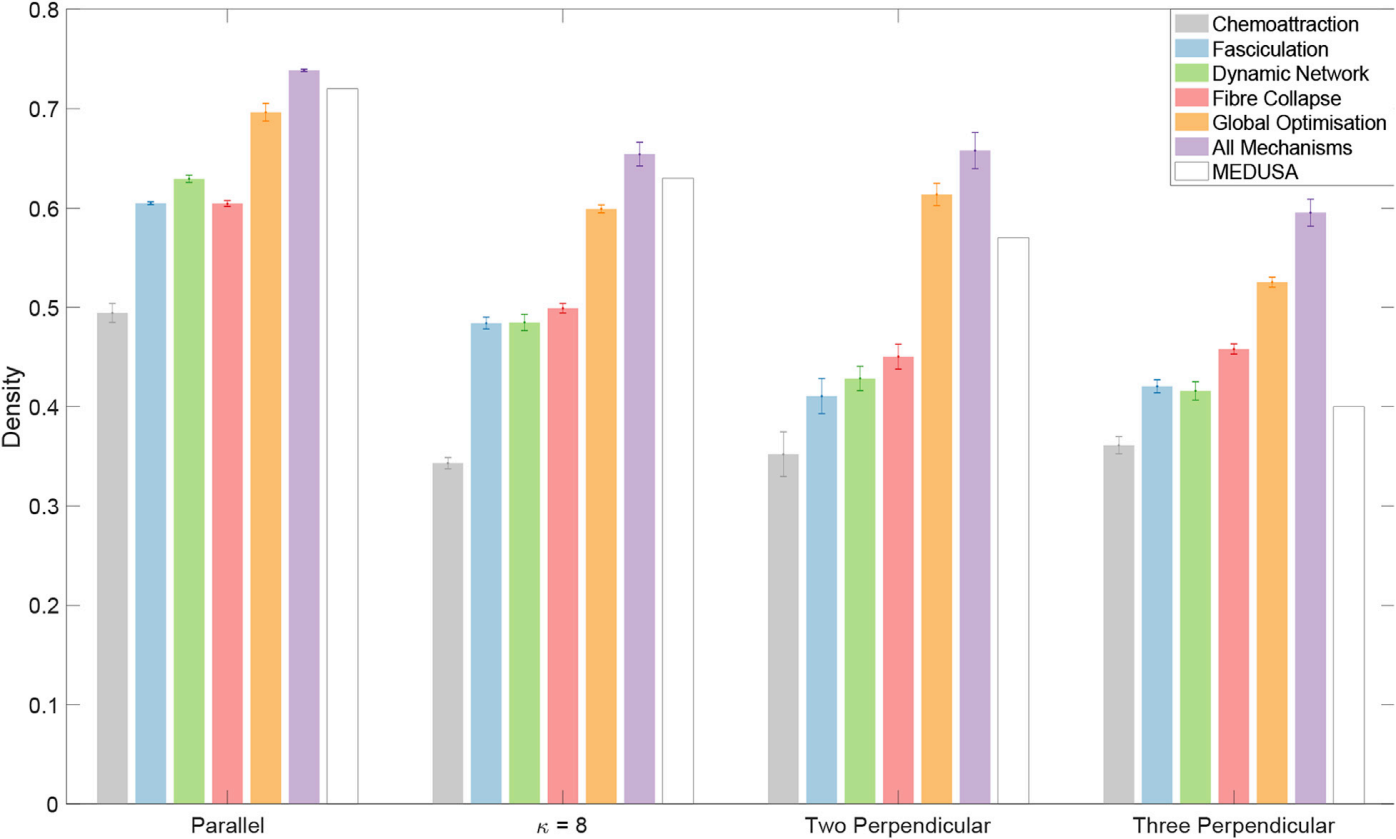
Demonstration of the impact of each biological growth mechanism on the density achievable with ConFiG. Each bar shows the mean density for each proposed mechanism, error bars show ​± ​standard error on the mean. MEDUSA values are estimated from Fig. 14 in Ginsberger et al. (Ginsburger et al., 2019).

Each of the proposed biological mechanisms enabled ConFiG to generate phantoms with increased density over the minimal case of chemoattraction only, as is shown in Fig. 10. Global optimisation resulted in the largest improvement, 17–24%, consistently giving a large improvement. Other improvements performed better for specific phantom configurations. For instance, fasciculation and the dynamic network produced only modest improvements in crossing fibre configurations (4–6%), but performed well in the single bundle cases (11–14%). Fibre collapse was particularly effective in the three perpendicular case, offering 10% improvement.

When combining all of the proposed mechanisms together, the achievable density is higher than any of the improvements individually. This improved performance is comparable to the state of the art, MEDUSA (Ginsburger et al., 2019), with particularly good performance relative to MEDUSA in the crossing fibre configurations.

**Fig. 11:**
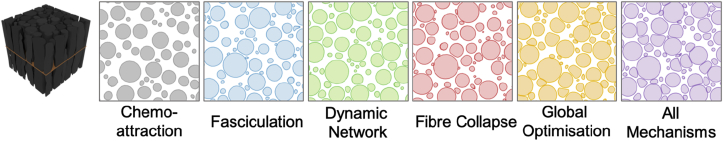
Virtual histology demonstrating the impact of biologically inspired mechanism on the final phantom created for one of the parallel phantoms tested. This visually demonstrates the improvement in density. Leftmost image shows the phantom generated with all mechanisms in 3D and the cutting plane used to produce the virtual histology.

**Fig. 12:**
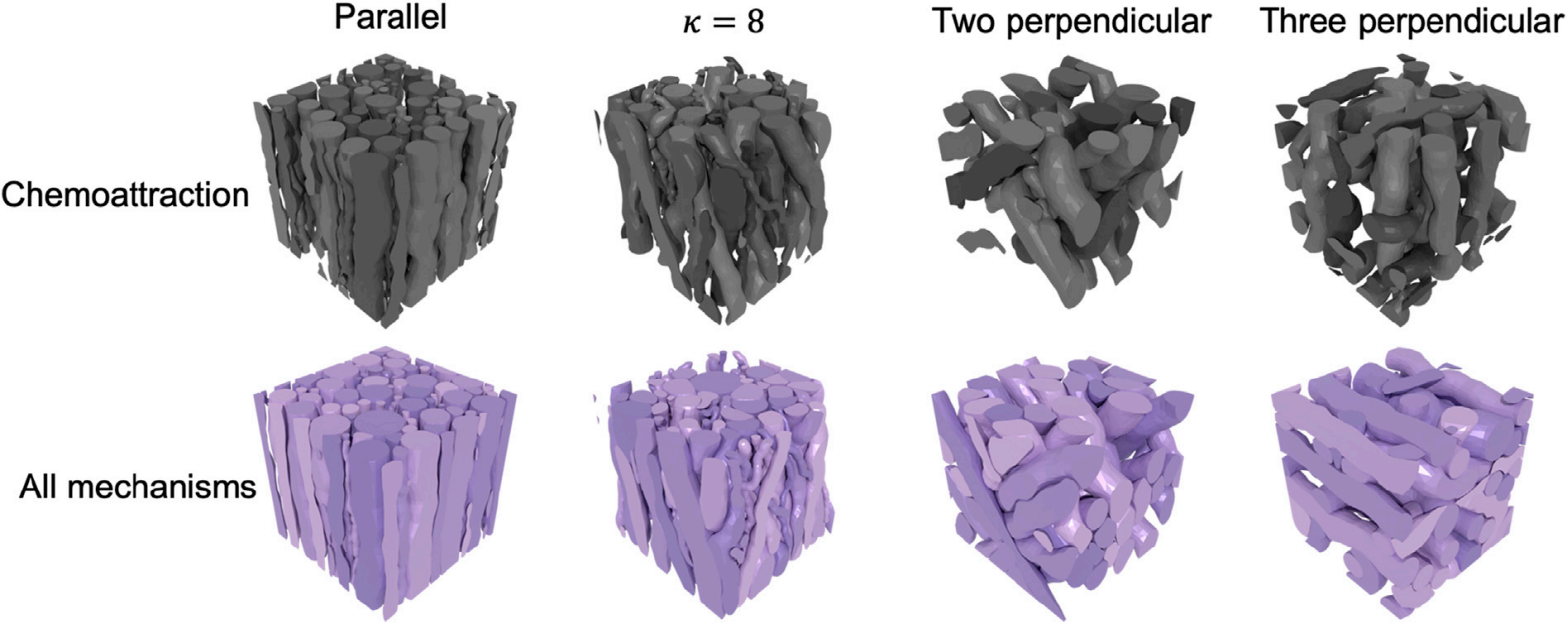
Demonstration of the improvement in density achieved when using all mechanisms in ConFiG compared to the minimal implementation using only chemoattraction. Colours chosen to match Fig. 1.

This improvement in density can be appreciated visually in Fig. 11 which demonstrates virtual histology of a parallel fibre phantom for each of the mechanisms. Additionally, Fig. 12 visually shows the difference in density of the phantoms in 3D between the minimal case of chemoattraction and all biological mechanism for each fibre configuration.

**Fig. 13:**
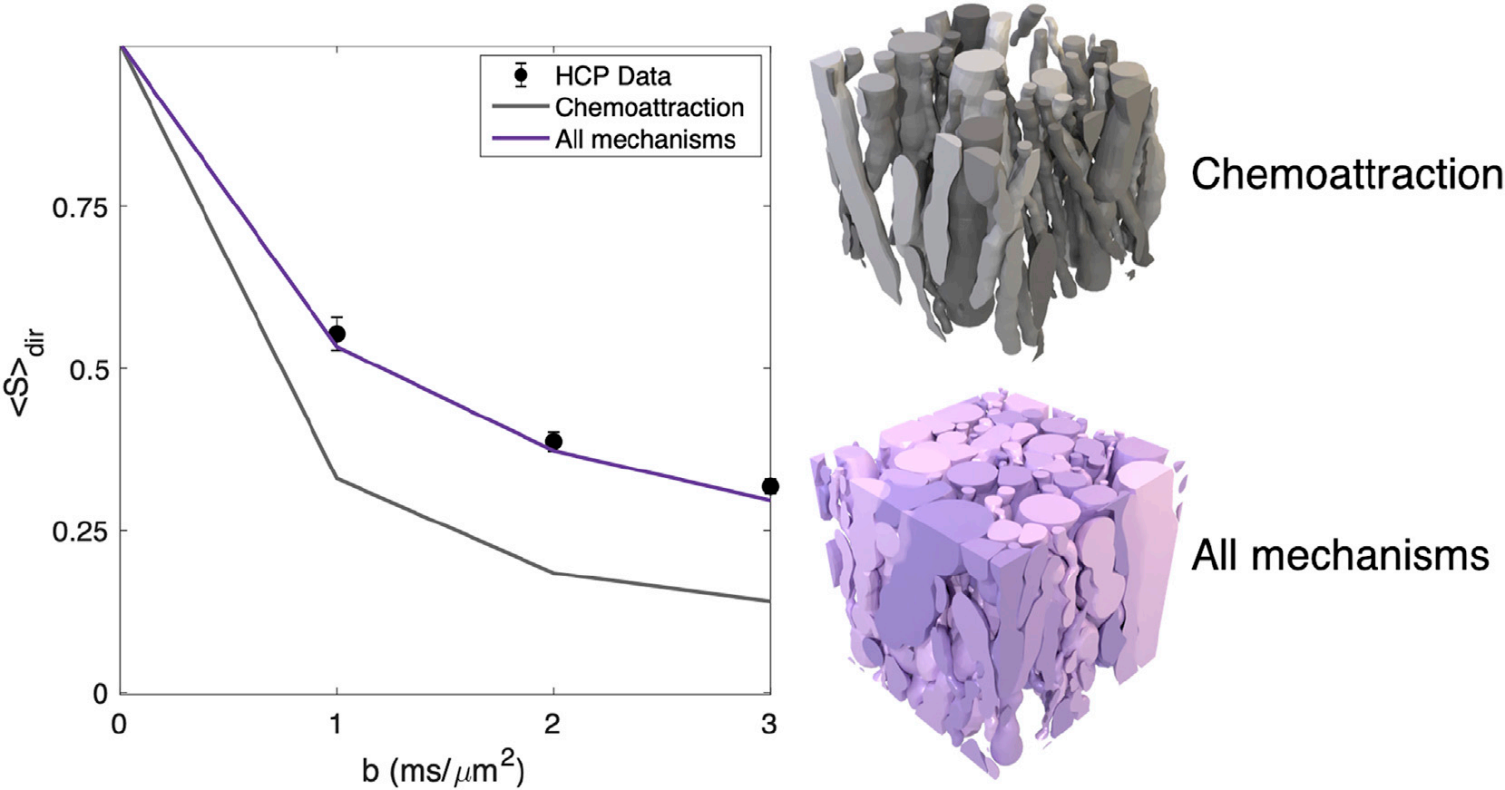
Left: Direction averaged signal attenuation for real HCP data (± standard deviation over ROI) and simulated data from the minimal ConFiG implementation using only chemoattraction and using all growth mechanisms ConFiG showing that ConFiG can produce realistic dMRI signals. Right: The original and improved ConFiG phantoms used to generate the signal on the left. Simulations performed with 10^5^ spins, 2000 timesteps and HCP measurement scheme (Sotiropoulos et al., 2013a). Diffusivity set to 2.0 $\mu m^{2}/ms$, chosen to be consistent with previously reported values (Hall and Alexander, 2009; Nilsson et al., 2009; Rensonnet et al., 2017).

The improvement in the density of phantoms leads to a much more realistic simulated diffusion MRI signal as demonstrated in Fig. 13. The root mean square error to the real data is reduced by 10 times when using improved ConFiG.

### 4.2 Microstructural measures and virtual histology

**Fig. 14:**
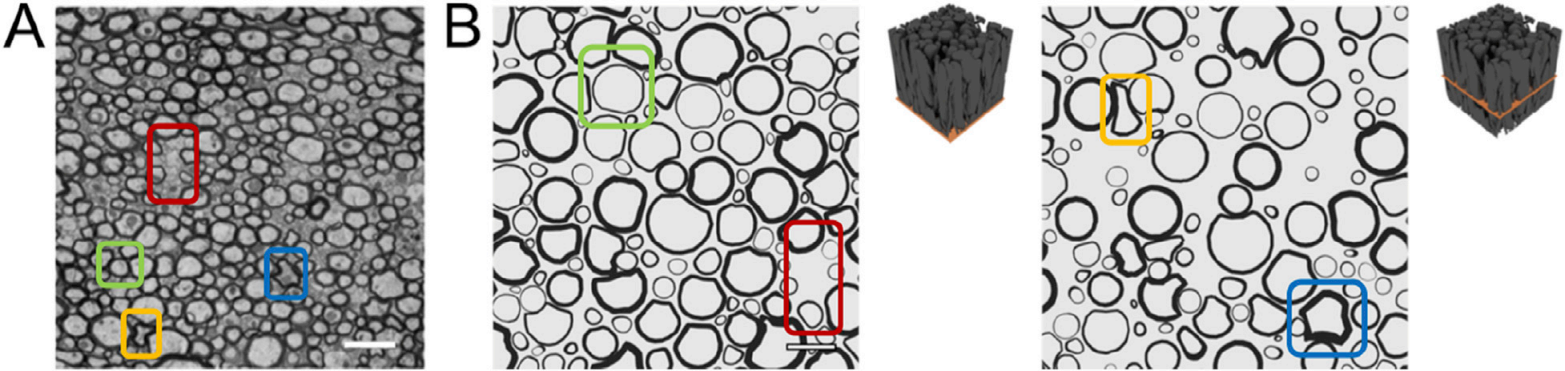
Comparison of real and virtual histology. A) Light microscopy of rat ventromedial WM in thoracic spinal cord. Reproduced from (Baxi et al., 2015), scale bar 2 ​μm. B) Two virtual histological slices from a ConFiG generated phantom. Phantoms are rendered to have similar colours to electron microscopy studies. The exact contrast and fibre bundle configurations are different between the real and virtual tissues, but the general morphology of the myelinated axons are captured well using ConFiG as highlighted by corresponding boxes. Yellow and Blue: axons severely deformed between other axons. Red: Pockets of empty space forming. Green: Largely circular axon surrounded by other axons deforming around it. Scale bar 2 ​μm.

**Fig. 15:**
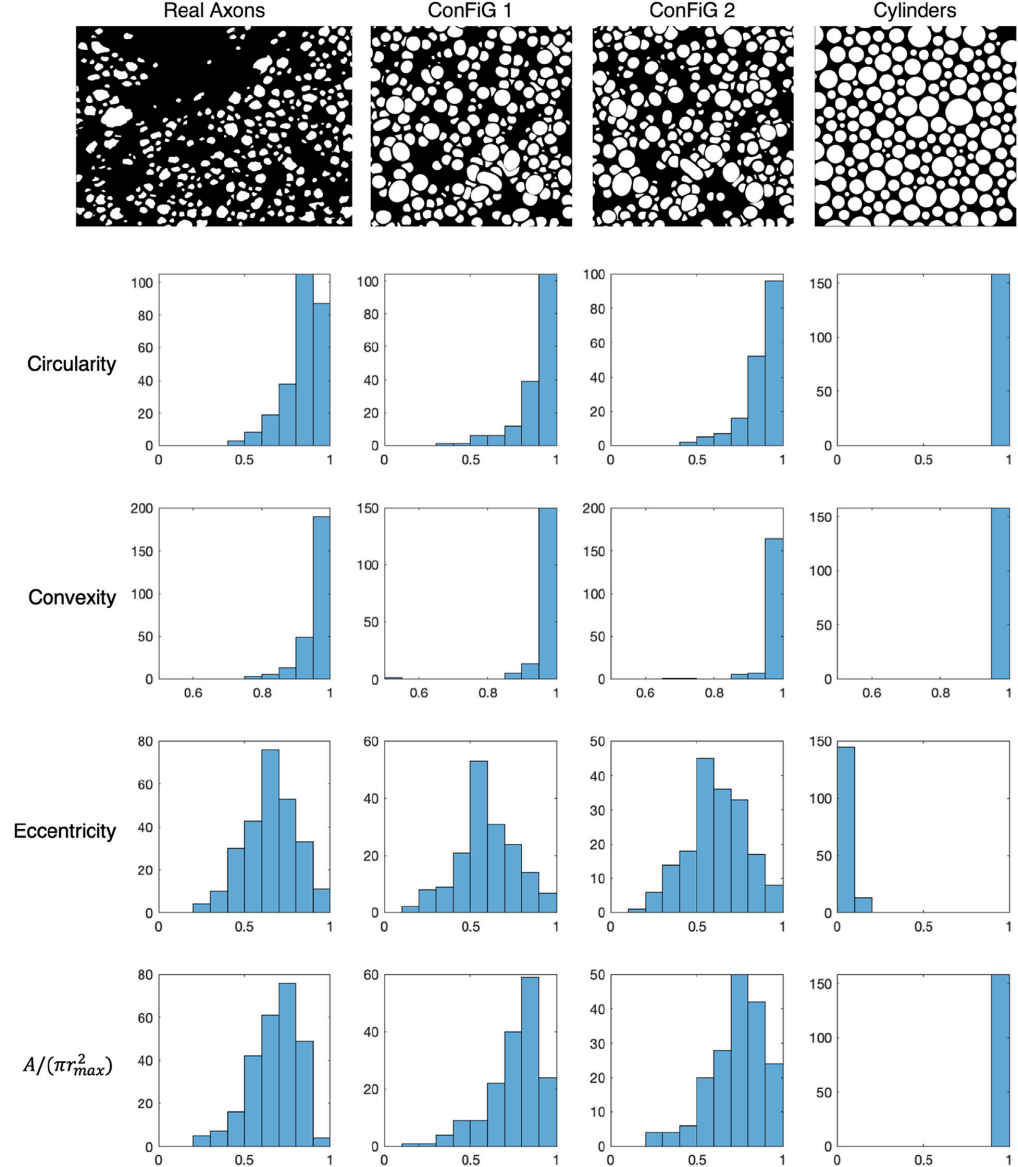
Slice wise morphological metrics calculated for real axons, ConFiG phantoms and parallel cylinders. Across each of the metrics, ConFiG produces much more realistic distributions than the cylinder phantom. Some of the cylinders have a non-zero eccentricity, but this arises since the metrics are calculated from binary images where the pixelated circles may appear to not be perfectly circular.

**Fig. 16:**
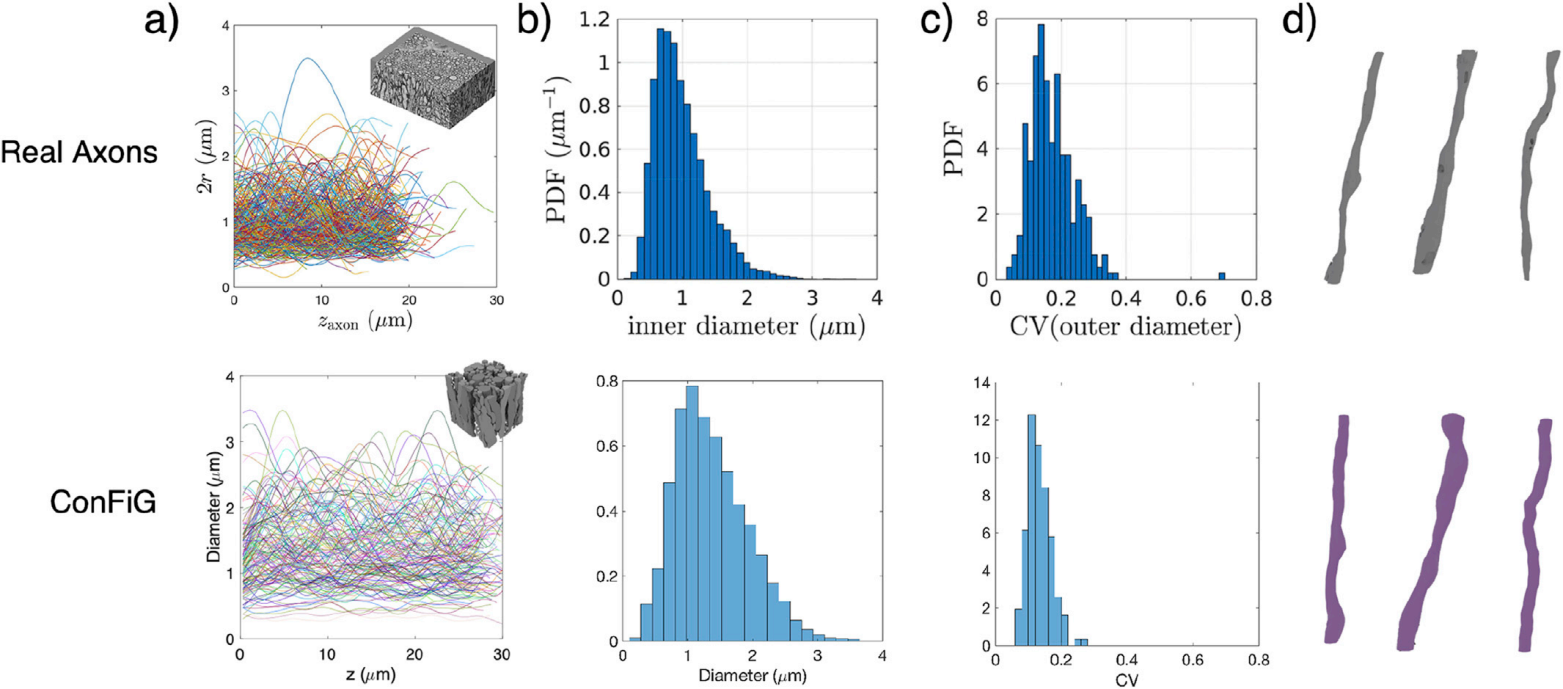
a) Along fibre diameter variation in ex vivo mouse corpus callosum, reproduced from (Lee et al., 2019) compared to along axis diameter variation in the phantom inset demonstrating the ability of ConFiG to generate realistic microstructure. b) Histograms of the inner diameter of axons from (Lee et al., 2019) and diameter of ConFiG axons. c) Coefficient of variation along axons for real and ConFiG axons and d) Three example fibres reconstructed from the EM data used by Lee et al. to make a). d) Three example ConFiG fibres selected for similarity to the EM examples.

**Fig. 17:**
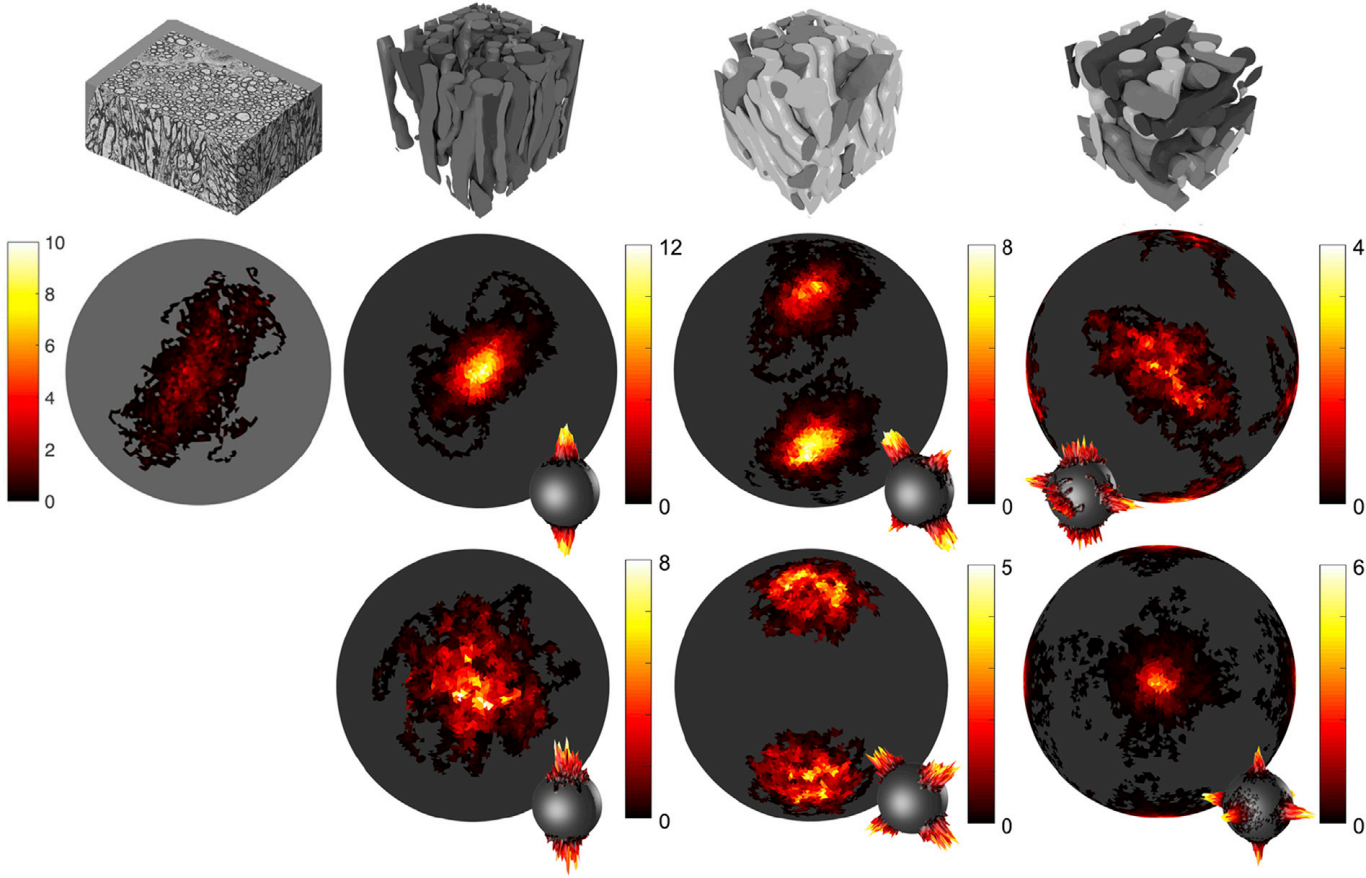
Dispersion profiles for EM data and a series of numerical phantoms. Top row: EM data used to generate OD profile, reproduced from Lee et al. (Lee et al., 2019) and three ConFiG phantoms with one, two and three crossing bundles (each crossing bundle coloured a different shade of grey). Middle row: OD profile for real EM data and OD profiles corresponding to ConFiG phantoms above, generated using an elliptically symmetric dispersion. Bottom row: Three OD profiles generated from ConFiG phantoms generated using isotropic orientation dispersion. Colormap has units of steradians^−1^.

The microstructural morphology generated using ConFiG is comparable to results from real data as demonstrated in Fig. 14, Fig. 15, Fig. 16, Fig. 17. Fig. 14 demonstrates virtual histology of a ConFiG phantom alongside a real EM image from mouse corpus callosum (Baxi et al., 2015). The exact microstructural features, such as diameter distribution, as well as the EM contrast do not exactly match between ConFiG and the real data. However, ConFiG is able to capture the general morphology or real axons as highlighted in Fig. 15, Fig. 16. In particular, ConFiG is able to capture complex fibre cross-sections such as in the case of fibres squashed into small spaces. This is the first model of white matter able to handle complex fibre cross-sections such as this to our knowledge.

Video 2 shows a series of sequential slices through a ConFiG substrate containing two crossing bundles of fibres, demonstrating the non-circular cross-sections generated by ConFiG. These complex cross-sections are not explicitly imposed during growth but arise as a result of the close packing of axons and the meshing procedure used in ConFiG.

Supplementary video related to this article can be found at https://vimeo.com/402472645 or https://doi.org/10.1016/j.neuroimage.2020.117107

The following is the supplementary data related to this article:

Video 2 Sequential virtual histology slices of a ConFiG phantom containing two crossing fibre bundles alongside the phantom in 3D with cutting plane indicated. Each fibre is given a unique colour to aid in differentiation and tracking of individual fibre. See also https://vimeo.com/402472645.

ConFiG morphological metrics calculated slice-wise on the virtual histology correspond much more closely to real axons that the same metrics calculated for parallel cylinders, as shown in Fig. 15. While cylinders produce a delta function at one extreme of each metric, ConFiG phantoms produce much closer distributions to the real data. Supplementary Fig. 2 shows each of these slices coloured by their morphological metrics.

The diameter distribution of a ConFiG substrate is compared to a reconstruction from real EM data (Lee et al., 2019) in Fig. 16. ConFiG is able to capture the general profile of axonal variations well, with the overall shape of the diameter distribution matching well. The distribution of the coefficient of variation along ConFiG axons is slightly narrower with a smaller mean than real axons, though these discrepancies may be alleviated with a different choice of input parameters to ConFiG.

ConFiG is also able to generate orientation distributions comparable to real tissue as shown in Fig. 17. The orientation dispersion is introduced to ConFiG phantoms using the elliptically symmetrical angular Gaussian (Paine et al., 2018) to best approximate the EM data and also using isotropic Watson distributed directions to demonstrate the flexibility of ConFiG.

### 4.3 Relationship between input and output morphology

The morphology of ConFiG phantoms matches the input morphology well, as shown in Table 2. Whilst the input and output $\mu_{\theta }$ and $\sigma_{\theta }$, do not match exactly, the values are close and increasing the input $\mu_{\theta }$ and $\sigma_{\theta }$ also increases the output $\mu_{\theta }$ and $\sigma_{\theta }$. Additionally, the output density generally matches the input target density well, achieving higher densities than MEDUSA for the same angular dispersion. Supplementary Table 1 shows the same experiment run with a target density of 60% to demonstrate ConFiG’s performance at lower densities.

**Table 2:** Comparison between input microstructural parameters and the microstructure measured in the resulting ConFiG phantoms. For each phantom, an input target density, ρ, of 75% was used with each phantom having a different value of κ used in the Watson distribution. Each κ is associated with a target $\mu_{\theta }$ and $\sigma_{\theta }$, the mean and standard deviation of the angle away from the main bundle direction. Angles reported in degrees.

Input κ	Input ρ	Output ρ	Target $\mu_{\theta }$	Output $\mu_{\theta }$	Target $\sigma_{\theta }$	Output $\sigma_{\theta }$	Output no. fibres
8	75%	70.6%	19.60	17.46	11.32	9.92	104
10	75%	73.4%	17.11	16.47	9.62	9.43	128
15	75%	73.4%	13.60	13.93	7.37	8.75	132
20	75%	70.7%	11.68	12.69	6.23	7.88	133
30	75%	72.0%	9.45	11.60	5.02	6.60	152
50	75%	73.6%	7.26	9.36	3.83	5.51	146
100	75%	74.9%	5.10	7.75	2.68	4.23	157

These phantoms took an average of 6 ​h to grow plus an average of 20 ​min for the meshing and microstructural measurement procedure, using 9.4 ​GB of RAM on average. These values give an estimate of the time taken to generate a typical ConFiG phantom, though it is strongly dependent on user inputs (number of nodes in the network etc.).

### 4.4 Diffusion MRI simulation

**Fig. 18:**
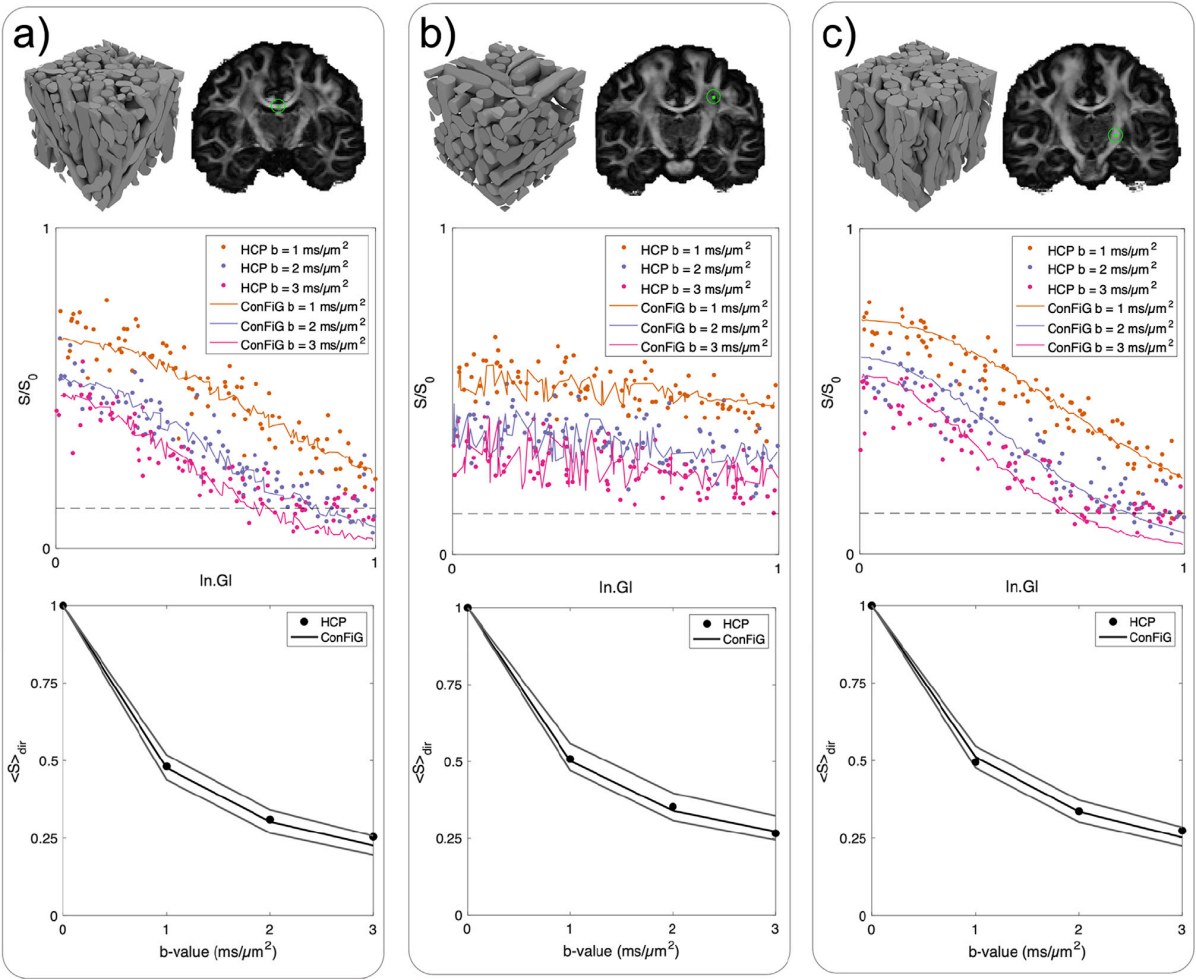
Comparison of diffusion MRI simulations and real data from three different brain regions: a) a voxel in the midbody of the corpus callosum, with phantom with volume fraction 55% and mean orientation from z 25°. b) a voxel in which there are three crossing bundles, with phantom of three crossing bundles with volume fraction 50% and c) a voxel in the internal capsule, with phantom with volume fraction 58% and mean orientation from z 22°. Top row shows the ConFiG phantom and corresponding WM voxel. Middle row shows the direction dependent signal for ConFiG (lines) and HCP data (dots). Bottom row shows the direction averaged signal. Black lines correspond to phantom in top row. Grey lines are signal from phantoms with the same orientation distribution as the black line in each plot but different densities to show that ConFiG has the flexibility to generate a wide range of realistic signals. Simulations performed with 10^5^ spins, 2000 timesteps, diffusivity 2.0 $\mu m^{2}/ms$ and HCP measurement scheme (Sotiropoulos et al., 2013a).

Simulated data from ConFiG substrates match real dMRI data well, as shown in Fig. 18. The direction averaged signal matches well in each case, in particular, for the corpus callosum and three crossing phantoms, the simulated signal matches the real signal closely. The b ​= ​3 ​ms/μm^2^ signal in the internal capsule and corpus callosum is lower in simulation than in real data. This is to be expected however because as |n.G| approaches 1, the signal reaches the noise floor and the noise-free simulations fall below the measured data. Supplementary Fig. 3 shows the difference between the simulated and measured signal in 3D.

## 5 Discussion

ConFiG is shown to produce substrates with microstructural properties comparable to real white matter, both in terms of measures derived from histology (i.e., electron microscopy) and in terms of the diffusion MRI signal.

ConFiG is shown to produce WM numerical phantoms with state-of-the-art performance. The amount of real data containing 3D microstructural morphology information available to compare to is limited, so we have only compared to one sample in this study. Whilst limited, this shows that ConFiG is able to produce realistic microstructure by following simple biologically inspired growth rules.

Fig. 15 demonstrates that ConFiG phantoms are able to create fibre morphologies that match real axons much more closely than previous methods based on cylinders. Whilst some of the features such as eccentricity may be achievable with cylinders oriented obliquely to the cutting plane, ConFiG phantoms capture morphological features that are otherwise impossible with cylinders such as convexity less than one.

Whilst the input morphological priors do not necessarily correspond to the morphology of the resulting ConFiG phantom, Table 2 shows that even for relatively high orientation dispersion and density, this effect is small. Even so, for use in further analysis, microstructural measures such as orientation dispersion and density should be calculated based on the resultant phantom, rather than taking the input microstructural parameters.

A related property of ConFiG is that the growth algorithm strongly depends on the growth network, meaning that the resulting phantom for the same input fibre configuration will be different for different network choices. This is alleviated to an extent by using the dynamic network introduced here, however the phantom will still be dependent on the initialisation of the network. The dependence appears to be relatively minor as is demonstrated by the small standard errors on the mean density shown in Fig. 10 across the five repetitions.

The diffusion MRI simulations shown in Fig. 18 demonstrate the ability of ConFiG to generate phantoms which reproduce real diffusion MRI data well. These simulations, however, are just three examples of ConFiG phantoms and corresponding simulations. Using NODDI as input to ConFiG means that the resulting phantoms have sensible morphologies and are shown to generate signals that match the real tissue well, though there may be other configurations that can better reproduce the signal. As an example, the b ​= ​3 ​ms/μm^2^ signal from the internal capsule is higher at low |n.G| in the simulated versus the real data (Fig. 18c). One explanation of this is that the phantom generated does not have microstructure accurately representing this region, for instance the phantom may have too little dispersion caused by ConFiG underrepresenting the target orientation dispersion, as seen at low κ in Table 2.

It may be possible to find a better matching phantom using a computational modelling approach such as that proposed in (Nedjati-Gilani et al., 2017), however the simulations presented are sufficient to demonstrate a proof-of-concept that ConFiG can be used to generate realistic simulated dMRI data.

### 5.1 Limitations and future work

One limitation of ConFiG is that the algorithm relies on the space being sufficiently densely sampled by the growth network. This can require a large number of nodes for a large phantom, becoming prohibitively memory expensive. The dependence of the resulting phantom on the density of network nodes can be addressed by growing the fibres in small subregions local to the head of the fibres rather than the whole space at once. For instance, rather than filling the entire space of growth with nodes, it is possible to fill a small layer of the space with points and then grow layer by layer. In this way, it is possible to achieve a high density of nodes using fewer nodes than when covering the entire space.

One further potential limitation of ConFiG is that once a fibre has grown, it is static. The fibre will remain fixed in place and all other fibres will have to grow around it. One problem with this is that once the fibres are fixed, they may create pockets of inaccessible space which limits the space available for following fibres. Additionally, in real tissue, axons are flexible and non-rigid, meaning that it may be more realistic that growing fibres can push existing fibres out of the way to make more space for growth. A potential approach to ameliorate this would be to have an optimisation procedure during growth, similar to the global optimisation introduced in this work but optimising the shape of a fibre as it grows.

A limitation of the current study is that the simulations assume a single diffusivity for the intra and extracellular spaces and no permeability of the axonal membranes. Furthermore, effects such as T2 and magnetic susceptibility are ignored. These effects are a limitation of the simulator used rather than ConFiG, and work is planned to improve these aspects of the simulator for more realistic simulated signals.

Additionally, as mentioned above, this study only compares ConFiG to one EM sample of real tissue. Future work will also aim at more extensive validation of the digital phantoms generated using ConFiG, making comparison with larger EM dataset, including different WM configurations from different brain regions.

We will work towards decreasing the difference between the input and output morphological measures, particularly in complex situations, such as high orientation dispersion and crossing bundles. This can be addressed through the improvements to ConFiG mentioned here and also by improving the strategy for the generation of starting and target points for each fibre. For instance, currently it is not intuitive how starting and target points should be arranged to achieve a desired density in crossing regions of fibres.

One planned extension of ConFiG is to implement periodic boundary conditions in the growth network, enabling the generation of fully periodic phantoms. This would enable ConFiG phantoms to be generated in relatively small volumes and tiled for simulation, accelerating the process of generating a wide range of phantoms and the memory required to store each phantom.

The core growth algorithm for ConFiG relies on a set of starting and target points, a connected network of nodes and some rules defining the growth. As such, ConFiG is very flexible since the exact form of each of these components can be modified based on the application. One example of a simple modification that may be explored is the order of growth of the axons. Currently, in the absence of any clear biological precedent know to the authors, fibres grow in a random order, but it may be possible that there is a better order such as growing large diameter axons first, or central axons in a bundle growing first.

In this work, ConFiG is applied to the case of densely packed axons, without contributions from neuronal cell bodies or other processes. A planned future extension of ConFiG is to allow for the addition of glial cells such as astrocytes and oligodendrocytes (e.g. from real 3D reconstructions available for instance on http://neuromorpho.org or synthetically generated using generative models like in (Palombo et al., 2019)) to the extracellular space to make the virtual WM tissue more realistic.

Additionally, to further add to the realism of ConFiG phantoms, realistic myelin may be modelled, creating spiral layers wrapped around the axons (Brusini et al., 2019). Furthermore, intra-axonal structures such as mitochondria and microtubules may be added to investigate their contributions to the diffusion weighted signal.

A planned future application will be to use ConFiG to generate a wide range of phantoms with different microstructural features. These can then be used to create a computational model to estimate microstructural features directly from the diffusion MRI signal in an approach similar to previous works (Hill et al., 2019; Nedjati-Gilani et al., 2017; Palombo et al., 2018a, 2016; Rensonnet et al., 2018).

### 5.2 Applications beyond diffusion MRI

As mentioned in the introduction, axonal configuration impacts MR signals beyond dMRI. One potential avenue of exploration would be to investigate the impact of realistic axonal configurations on magnetic susceptibility in a similar way to Xu et al. (2018), extending their 2D simulations to use realistic 3D geometries generated in ConFiG.

The virtual histology presented in Fig. 14 shows an approximation of electron microscopy generated using ConFiG substrates. In this work, the purpose of this is to show that ConFiG is generating microstructurally realistic phantoms. For this reason, the virtual histology is simply produced by rendering images to have similar contrast to electron microscopy for comparison. It may be possible, however to generate more realistic electron microscopy images using a physically realistic electron microscopy simulator (Babin et al., 2010; Grella et al., 2003; Ophus, 2017) which may be used to train and test axon segmentation routines. This may be of particular use for cases of fibres parallel to the electron microscopy plane or crossing bundles which are typically difficult for 3D reconstruction and segmentation algorithms.

The 3D meshes generated by ConFiG are saved in the PLY format, a widely used format for storing meshes for many purposes. This means that the ConFiG phantoms may be used in other types of simulations such as polarized light imaging (Matuschke, 2019; Menzel et al., 2015) or molecular dynamics simulations using software such as MCell (https://mcell/.org) (Kerr et al., 2008; Stiles et al., 1996; Stiles and Bartol, 2001) or LAMMPS (http://lammps.sandia.gov) (Plimpton, 1997).

## 6 Conclusion

ConFiG enables the generation of realistic white matter numerical phantoms achieving state of the art fibre density whilst ensuring realistic microstructural morphology by following biologically motivated rules. This realistic microstructure is shown to generate realistic simulated diffusion MRI signals, opening up the possibility to use ConFiG to create a realistic computational model of WM microstructure.

ConFiG outputs fibre meshes which can be used for realistic diffusion MRI simulations or can be processed to produce virtual histological slices, allowing for further potential applications outside of diffusion MRI.

## Data and code availability

ConFiG code will be made available at https://rcallagh.github.io.

## CRediT authorship contribution statement

**Ross Callaghan:** Conceptualization, Methodology, Software, Investigation, Visualization, Writing - original draft. **Daniel C. Alexander:** Conceptualization, Writing - review & editing, Supervision, Resources, Funding acquisition. **Marco Palombo:** Conceptualization, Methodology, Writing - review & editing, Supervision. **Hui Zhang:** Conceptualization, Methodology, Writing - review & editing, Supervision, Resources, Funding acquisition.

## Declaration of competing interest

The authors confirm that there are no conflicts commercial or financial conflicts of interest affecting this work.
