# Supplementary material for: ConFiG: Contextual Fibre Growth to generate realistic axonal packing for diffusion MRI simulation
Source: Neuroimage. 2020 Oct 15;220:117107. doi: 10.1016/j.neuroimage.2020.117107 (PMC7903162; doi:10.1016/j.neuroimage.2020.117107)
Supplement: Multimedia component 3 [file mmc3.docx]

Supplementary Materials

Supplementary Figure 1 Axial diffusivity as a function of time using different metaball meshing thresholds. The threshold determines the level of the isosurface, a low threshold making the spheres behave more like hard spheres and a higher threshold smoothing nearby metaballs together. Lines show the median of 20 runs of the simulation with different seeds and the grey area shows the interquartile range of the cylinder diffusivity.


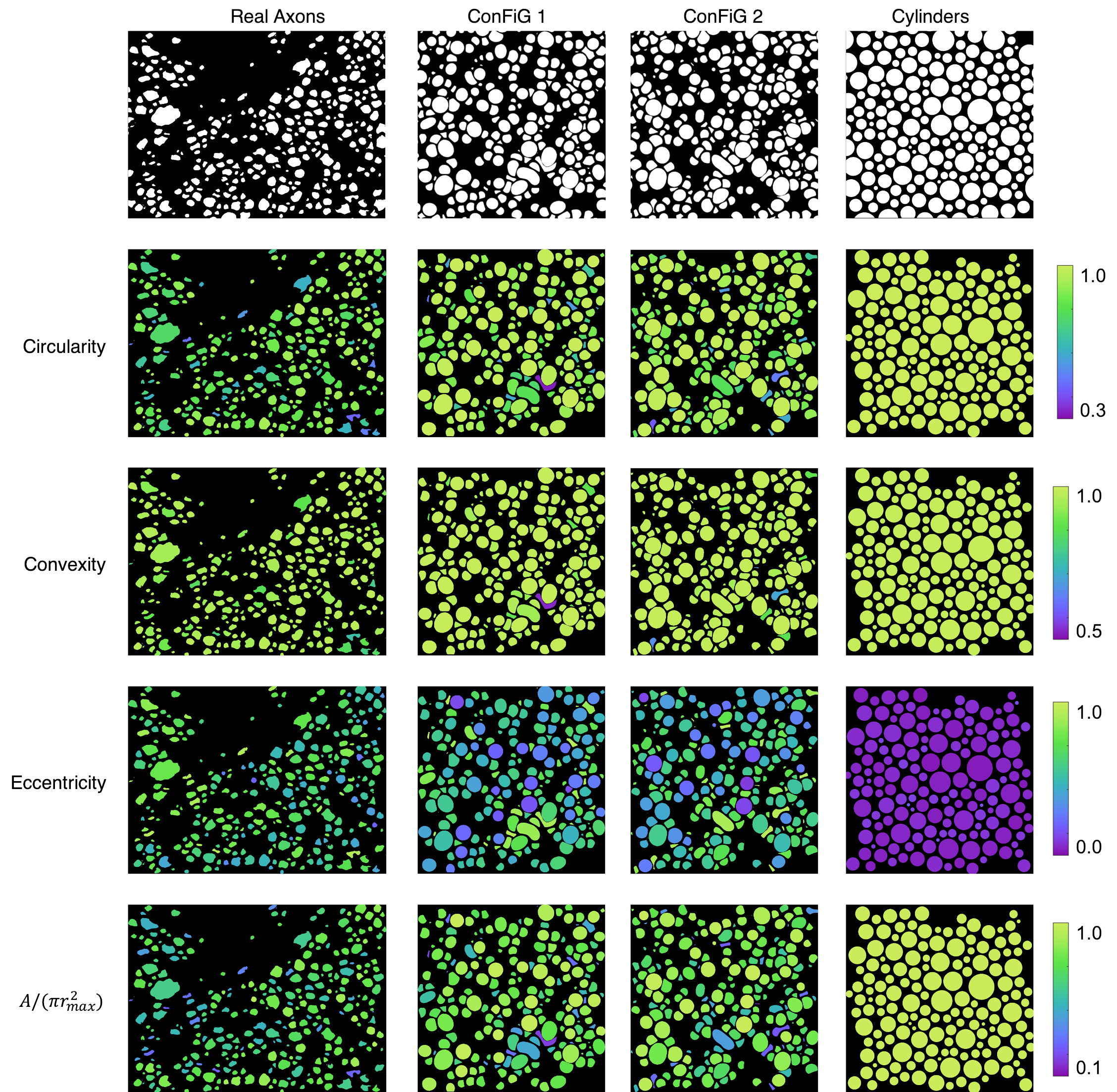


Supplementary Figure 2 Histological segmentations of axons coloured by morphological metrics for real axons, ConFiG phantoms and a cylinder phantom.


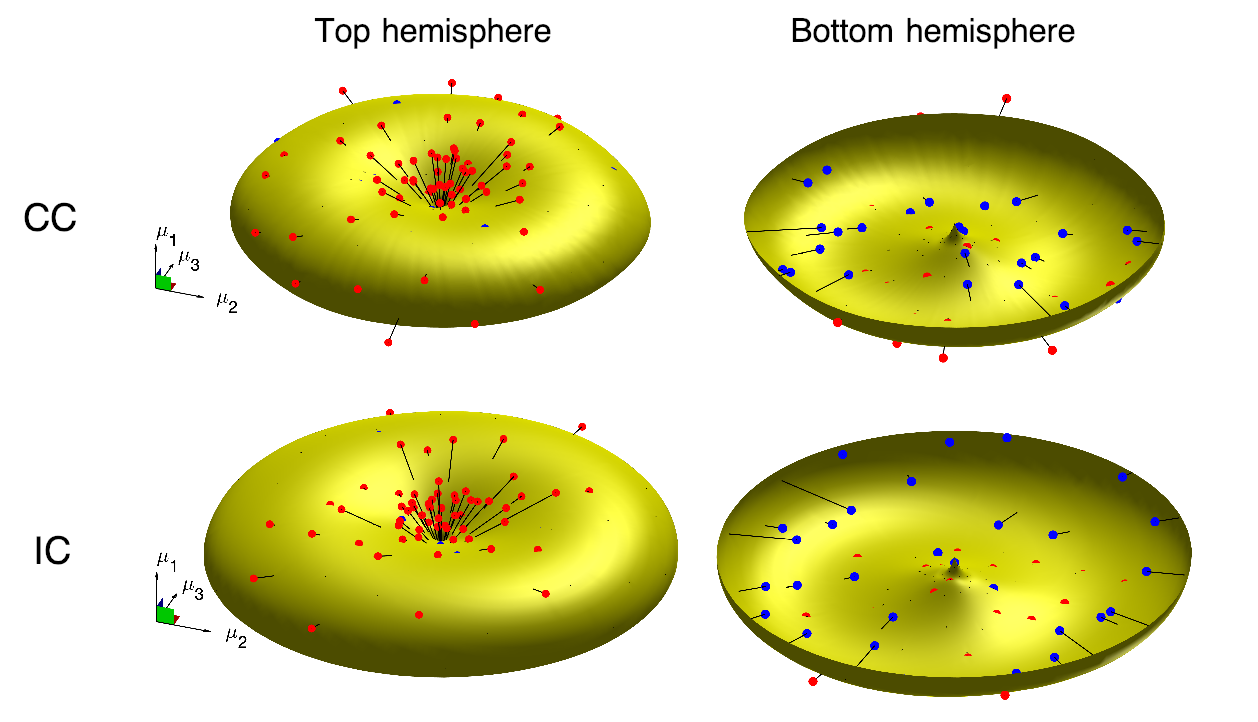


Supplementary Figure 3 3D plots of difference between measured and simulated signal for b = 3ms/µm^2^. Surface is 6th order SH representation of the simulated signal. Points are measured data coloured such that red points have measured signal higher than corresponding simulated signal and blue points have signal lower than simulated signal. Lines connected to each point show distance to corresponding point on the simulated signal surface.

Supplementary Table 1 Comparison between input microstructural parameters and the microstructure measured in the resulting ConFiG phantoms. For each phantom, an input target density, $\rho$, of 69% was used with each phantom having a different value of $\kappa$ used in the Watson distribution. Each $\kappa$ is associated with a target $\mu_{\theta}$ and $\sigma_{\theta}$, the mean and standard deviation of the angle away from the main bundle direction. Angles reported in degrees.

| Input $\kappa$ | Input $\rho$ | Output $\rho$ | Target $\mu_{\theta}$ | Output $\mu_{\theta}$ | Target $\sigma_{\theta}$ | Output $\sigma_{\theta}$ | Output no. fibres |
| --- | --- | --- | --- | --- | --- | --- | --- |
| 8 | 60 | 60.6 | 19.60 | 16.60 | 11.32 | 8.81 | 66 |
| 10 | 60 | 59.8 | 17.11 | 16.77 | 9.62 | 10.08 | 74 |
| 15 | 60 | 61.4 | 13.60 | 14.35 | 7.37 | 7.71 | 77 |
| 20 | 60 | 59.9 | 11.68 | 13.89 | 6.23 | 8.15 | 81 |
| 30 | 60 | 57.6 | 9.45 | 10.02 | 5.02 | 5.99 | 73 |
| 50 | 60 | 59.1 | 7.26 | 8.43 | 3.83 | 5.02 | 81 |
| 100 | 60 | 61.2 | 5.10 | 7.31 | 2.68 | 4.04 | 88 |
